# Supplementary material for: Policymakers’ concerns linking tobacco and Indigenous communities in India: A qualitative analysis of parliamentary questions (1952–2022)
Source: PLOS Glob Public Health. 2026 Feb 24;6(2):e0004601. doi: 10.1371/journal.pgph.0004601 (PMC12931741; doi:10.1371/journal.pgph.0004601)
Supplement: Attachment — Submitted filename: PGPH-D-25-00953_R1 (1).pdf [file pgph.0004601.s002.pdf]

PLOS Global Public Health

Policymakers' concerns linking tobacco and Indigenous communities in India: a qualitative analysis of parliamentary questions (1952-2022)

--Manuscript Draft--

**Manuscript Number:**	PGPH-D-25-00953R1
**Article Type:**	Research Article
**Full Title:**	Policymakers' concerns linking tobacco and Indigenous communities in India: a qualitative analysis of parliamentary questions (1952-2022)
**Short Title:**	Tobacco and Indigenous communities in India
**Corresponding Author:**	Upendra Bhojani<br>Institute of Public Health Bengaluru<br>Bangalore, Karnataka INDIA
**Order of Authors:**	Shilpi Sikha Das, MPhil, PhD
	Upendra Bhojani, MPH, PhD
	Prashanth Nuggehalli Nuggehalli Srinivas, MPH, PhD
**Keywords:**	Indigenous communities; Adivasis; India; Tobacco; Health policy; parliament; policymaker
**Abstract:**	Tobacco use and associated disease burden remains high among Indigenous communities in India. Despite an overall decline in tobacco consumption over the last few decades, the social disparities in tobacco use have widened with Indigenous communities experiencing the least decline. Existing tobacco control policies lack specific considerations for Indigenous communities. Hence, as part of a broader research initiative focusing on the health of Indigenous communities, we explored how parliamentarians in India have framed their concerns about tobacco with reference to Indigenous communities over time.<br>We sourced digital transcripts of exchanges (parliamentarians asking questions and ministers responding to them) as part of the Question Hour sessions in the lower- and upper- houses of the Indian parliament between 1952 and 2022. We used thematic content analysis for 301 transcripts that linked tobacco and Indigenous communities.<br>Overall representation of Indigenous communities in the Indian parliament remains very limited. The major concerns expressed by parliamentarians included (1) occupational health hazards and inadequate access to healthcare services faced by members from Indigenous communities working as bidi makers and tendu leaf collectors; (2) unfair wages and exploitative work conditions for these workers; and (3) perceived negative impact on tobacco-linked livelihoods resulting from trade and investment related policies in the tobacco sector. Parliamentarians did not raise issues related to high tobacco use and tobacco-related harms among Indigenous communities in general, nor did they discuss about the negative impact of commercial tobacco production on forests that are central to the lives of Indigenous communities.<br>Public health research and policy engagement efforts need to acknowledge the complex and multiple intertwining links between tobacco (industry) and Indigenous communities. There is a need to sensitise elected representatives on the health and environmental impacts of tobacco while addressing the prevailing exploitation of workers from Indigenous communities in precarious tobacco supply chains and providing viable alternative livelihoods.
**Additional Information:**	
**Question**	**Response**
**Financial Disclosure**<br><br>Enter a financial disclosure statement that describes the sources of funding for the	This work was supported by the DBT Wellcome Trust India Alliance CRC grant (IA/CRC/20/1/600007 to PNS; IA/CRC/20/1/600007 to UB) and the DBT Wellcome Trust India Alliance (clinical and public health) Senior Fellowship (IA/CPH/22/1/506533 to UB). The funders had no role in study design, data collection and analysis, decision to publish, or preparation of the manuscript.

Powered by Editorial Manager® and ProduXion Manager® from Aries Systems Corporation

work included in this submission and the role the funder(s) played. This includes grants and any commercial funding of the work or authors.<br><br>This statement will be typeset if the manuscript is accepted for publication.<br><br>*Please review the submission guidelines and the instructions link below for detailed requirements and guidance.*	
**Competing Interests**<br><br>On behalf of all authors, disclose any competing interests that could be perceived to bias this work.<br><br>This statement will be typeset if the manuscript is accepted for publication.<br><br>*Please review the instructions link below and PLOS Global Public Health's competing interests policy to determine what information must be disclosed at submission.*	The authors have declared that no competing interests exist.
**Data Availability**<br><br>Before publication, Authors are required to make fully available and without restriction all data underlying their findings. Please see our PLOS Data Policy page for detailed information on this policy.<br><br>A **Data Availability Statement**, detailing where the data can be accessed, is required at first submission. Insert your Data Availability Statement in the box below.<br><br>Please see the data reporting section of our submission guidelines for instructions	Apart from the data contained in the manuscript, the transcripts of parliamentary questions (and answers) are available in public domain through web portals of the lower house (https://sansad.in/ls/questions/questions-and-answers) and the upper house (https://sansad.in/rs/questions/questions-and-answers) of Indian parliament.

Powered by Editorial Manager® and ProduXion Manager® from Aries Systems Corporation

on what you need to include in your Data Availability Statement.<br><br>This statement will be typeset if the manuscript is accepted for publication.<br><br>*PLOS allows rare exemptions to address legal and ethical concerns. If you have legal or ethical restrictions, please use the box below to detail these in full sentences for the Journal team to consider.*	

Powered by Editorial Manager® and ProduXion Manager® from Aries Systems Corporation

Manuscript

Click here to access/download;Manuscript;maintext_bhojani_April 25.docx

Parliamentarians' concerns about tobacco and Indigenous communities in India: a qualitative analysis of parliamentary questions (1952-2022)

Shilpi Sikha Das[1,2], Prashanth NS[1], Upendra Bhojani[1,2*]

[1]Centre of Adivasi Health, Institute of Public Health, Bengaluru, Karnataka, India

[2]Centre for Commercial Determinants of Health, Institute of Public Health, Bengaluru, Karnataka, India

[*]Corresponding author

Email: upendra@iphindia.org

Abstract

Tobacco use and associated disease burden remains high among Indigenous communities in India. Despite an overall decline in tobacco consumption over the last few decades, the social disparities in tobacco use have widened with Indigenous communities experiencing the least decline. Existing tobacco control policies lack specific considerations for Indigenous communities. Hence, as part of a broader research initiative focusing on the health of Indigenous communities, we explored how parliamentarians in India have framed their concerns about tobacco with reference to Indigenous communities over time.

We sourced digital transcripts of exchanges (parliamentarians asking questions and ministers responding to them) as part of the Question Hour sessions in the lower- and upper- houses of the Indian parliament between 1952 and 2022. We used thematic content analysis for 301 transcripts that linked tobacco and Indigenous communities. Overall representation of Indigenous communities in the Indian parliament remains very limited. The major concerns expressed by parliamentarians included (1) occupational health hazards and inadequate access to healthcare services faced by members from Indigenous communities working as bidi makers and *tendu* leaf collectors; (2) unfair wages and exploitative work conditions for these workers; and (3) perceived negative impact on tobacco-linked livelihoods resulting from trade and investment related policies in the tobacco sector. Parliamentarians did not raise issues related to high tobacco use and tobacco-related harms among Indigenous communities in general, nor did they discuss about the negative impact of commercial tobacco production on forests that are central to the lives of Indigenous communities.

Public health research and policy engagement efforts need to acknowledge the complex and multiple intertwining links between tobacco (industry) and Indigenous communities. There is a need to sensitise elected representatives on the health and environmental impacts of

tobacco while addressing the prevailing exploitation of workers from Indigenous communities in precarious tobacco supply chains and providing viable alternative livelihoods.

Keywords

Indigenous communities; Adivasis; India; tobacco; health policy; parliament

Introductio[illegible]

The widespread tobacco consumption in India poses a huge public health challenge. Tobacco consumption accounts for about 1.94 million adult deaths annually in India apart from a significant morbidity burden.(1) In India, tobacco consumption varies widely across social groups. The report of the Expert Committee on Tribal Health set up by the Government of India highlighted that while tobacco use among Indigenous communities varied across states, in general, it remained high among Indigenous communities.(2) It indicated that as per the National Nutrition Monitoring Bureau survey (2008-2009) covering nine Indian states, 35.9% of men and 6.3% of women in Indigenous communities (categorised as 'Scheduled Tribes' as per the Indian constitution) smoked tobacco.(2) Furthermore, 37.7% of men and 23.1% of women from Indigenous communities chewed tobacco.(2) The report indicated that the true prevalence of tobacco use among Indigenous communities might be higher as the nationally representative survey (National Family Health Survey) in 2005-2006 indicated the overall prevalence of tobacco use among men from Scheduled Tribe category to be 71.7%, much higher compared to men not belonging to Scheduled Tribes (56.3%).(2) Our earlier work (data unpublished) analysing the comparable rounds of the National Sample Survey revealed that such disparity in tobacco use existed in 1990 with people belonging to the Scheduled Tribe category experiencing the highest prevalence of tobacco use.(3) While the overall prevalence of tobacco use declined over the 1990-2012 period that we studied, the disparity across social groups widened with people in the Scheduled Tribe category experiencing the least decline in tobacco consumption.(3)

This implies that public health policies/programs have not equitably benefited all the social groups in India. While there have been several policy initiatives by the Government of India for reducing tobacco use(4) and these initiatives seem to be making a difference(5,6), there is no specific focus or considerations related to Indigenous communities. As part of the Centre for Training, Research and Innovation in Tribal Health, a multi-institutional research initiative, we have been researching substance use and haemoglobinopathies among Indigenous communities.(7) As part of this broader research agenda, we are conducting research on various aspects of substance use among Indigenous communities: from exploring sociocultural pathways of substance use among local communities to mapping

policy discourse related to substance use among Indigenous communities over time. As parliamentarians play a crucial role in shaping policy discourse and policymaking, including for tobacco control, we aimed to study how parliamentarians in India framed their concerns related to tobacco and Indigenous communities in India over time. Such understanding will inform how tobacco has been perceived and/or problematised concerning Indigenous communities in the Indian parliament with implications on tobacco control advocacy efforts with policymakers in India.

Methods

Study design and setting

We used a qualitative study wherein we analysed the digital transcripts of questions and answers that referred to tobacco and Indigenous communities in India as part of the parliamentary proceedings in both the houses of Indian parliament. India is a union of states with a representative parliamentary democracy and a bicameral legislature. We chose to source the digital transcripts of questions and answers happening as part of the Question Hour of the lower (*Lok Sabha*) and the upper (*Rajya Sabha*) houses of the Indian parliament. The Question Hour is a platform wherein concerned ministers respond to the prior-submitted questions by any members of parliament.(8,9) These questions can cover any aspects of government functioning and administration as well as raising concerns of their electorates. Hence, the idea is to promote government transparency and accountability to the electorate.(8,9) Our study focused on analysing questions/answers starting from the year 1952 (the year Question Hour was mooted, and digital transcripts are available from the web portals of the parliament) till the end of the year 2022.

Data collection

We searched the publicly available digital catalogues of parliamentary questions from both houses separately using a combination of search terms 'tobacco' and 'tribal' within the full text of the transcripts without any search filters. We used synonyms/substitutes of these terms (for tobacco: *bidi, beedi, biri, tendu, gutka, cigarette, hookah, hukka, kharra, gul, mishri, mawa, gudakhu, nastaar, khaini, chillum,* cigar, *and cheroot*; for tribal: *adivasi, adiwasi, and* indigenous) in all possible combinations. A total of 2556 transcripts were sourced with the oldest dating May 29, 1952, and the most recent dating July 26, 2022. Such a search strategy was arrived at after trying various searches to better understand how these search catalogues work (e.g., they do not recognise Boolean operators). The search results were exported into MS Excel spreadsheet and duplicates were removed using the relevant function. The first author (SSD) read all the transcripts in full and further excluded those that did not contain at least one of the two categories of search terms (tobacco and tribal) or had these terms occurring incidentally (often in answers) while the transcript had no substantive relevance to the subject of our study. Fig 1 provides details about the sourcing and screening outcomes. The decision about the exclusion of transcripts due to non-relevance to the study subject was taken through

discussion and consensus between the first (SSD) and the last (UB) authors. We analysed 301 transcripts for this study.

Fig 1 Selection of transcripts of parliamentary questions for analysis (1952-2022)

Data management and analysis

The transcripts were typically a few pages long. An MS Excel spreadsheet was used to create a dataset wherein each row represented a unique question (and answer) asked in the parliament. Various attributes related to that question were recorded (e.g., date when asked, parliamentarian/s asking the question, the parliamentary house, the ministry which was asked that question). Some of the questions were asked by more than one parliamentarian, and some of the parliamentarians had asked more than one question related to tobacco and Indigenous communities. Hence, another MS Excel spreadsheet was used to create a dataset wherein each raw represented a unique parliamentarian. Various attributes of that parliamentarian were recorded (e.g., gender, political party affiliation, constituency, and the state that the parliamentarian represented).

We used thematic content analysis using an interpretive lens to analyse transcripts. As this was an exploratory work with no precedent of similar inquiry (linking tobacco and Indigenous communities), the first author (SSD) used inductive coding of transcripts, guided by the overall question of 'how parliamentarians were framing their tobacco-related concerns in relation to the Indigenous communities?' rather than using any specific theoretical framework. She periodically discussed the coding pattern and the emerging themes she articulated based on coding with the last author (UB). Both these authors had formal training in qualitative research. The first author has worked with Indigenous communities for her doctoral and post-doctora work.(10,11) The last author has been researching tobacco control for about two decades including published work using parliamentary questions.(12) He has been collaborating with scholars working with Indigenous communities including some of the members from the Indigenous community. Both authors, through periodic discussions and iterations, agreed on the overall themes and explored the interrelations across themes. These findings are presented using themes and specific quotes from the data. At some point, both datasets were combined through unique IDs given to transcripts to describe the data using attributes of the parliamentarians (presented in charts/tabular format).

Results

About the parliamentarians

A total of 257 parliamentarians asked 301 questions over the 70-year period (1952-2022) linking tobacco and Indigenous communities. Most of them were in the lower house of parliament (76.7%) and were men (89.5%). Only 9.5% of these parliamentarians belonged to the Scheduled Tribe category. Over 60% of them belonged to the two major national political

parties: Indian National Congress accounting for 36.2% and Bharatiya Janata Party accounting for 24.1% of these parliamentarians. The remaining 40% of parliamentarians belonged to 31 different political parties including independent and nominated candidates. These parliamentarians represented constituencies across 28 states (including union territories and erstwhile jurisdictions) with Uttar Pradesh (15%), Andhra Pradesh (9%) and Maharashtra (8%) accounting for a third of these parliamentarians. Table 1 and Fig 2 provide details about select attributes of these parliamentarians.

Table 1 Parliamentarians asking questions about tobacco and Indigenous communities (1952-2022)

	**Parliament House**			**Gender**			**Social Group**		
**Political party**	***Lok Sabha*** **(Lower House)**	***Rajya Sabha*** **(Upper House)**	**Total**	**Men**	**Women**	**Total**	**Scheduled Tribe**	**Others**	**Total**
Congress	72	21	93	85	8	93	7	86	93
Bharatiya Janata Party	51	11	62	53	9	62	8	54	62
Independent and nominated candidates	6	4	10	9	1	10	0	10	10
All India Anna Dravida Munnetra Kazhagam	8	1	9	8	1	9	1	8	9
Communist Party of India (Marxist)	6	3	9	8	1	9	1	8	9
Telugu Desam Party	4	4	8	6	2	8	1	7	8
Shiv Sena	7	0	7	6	1	7	0	7	7
Biju Janata Dal	4	2	6	5	1	6	2	4	6
Samajwadi party	6	0	6	5	1	6	0	6	6
Dravida Munnetra Kazhagam	2	3	5	5	0	5	0	5	5
Others (24 political parties combined with each having less than 5 parliamentarians)	31	11	42	40	2	42	5	37	42
**Total**	**197 (76.7%)**	**60 (23.3%)**	**257**	**230 (89.5%)**	**27 (10.5%)**	**257**	**25 (9.7%)**	**232 (90.3%)**	**257**

Fig 2 Parliamentarians (state-wise) asking questions linking tobacco and Indigenous communities (1952-2022)

Parliamentarians' concerns

We now describe parliamentarians' concerns about tobacco in relation to Indigenous communities in India using narratives and example questions under specific themes that emerged from analysis of the transcripts. Table 2 summarises these concerns by mentioning specific themes and issues within those themes.

Table 2: Themes defining parliamentarians' concerns about tobacco and Indigenous communities in India

Themes defining parliamentarians' concerns	Specific aspects/issues within each theme
Health concerns	Specific health issues affecting bidi workers<br>● Tuberculosis<br>● Hunger<br>Access to healthcare services by workers and their families<br>● Hospitals for bidi workers<br>● Basic healthcare services for *tendu* leaf collectors<br>● Coverage (workers and their families) and implementation of the publicly funded health insurance scheme (RSBY)
Labour welfare concerns	Bidi worker's welfare fund<br>● Contributions by employers (bidi industry) towards bidi workers' welfare fund<br>● Effective allocation and utilisation of such funds by governments towards workers' welfare<br>Employees Provident Fund<br>● Facilitating enrolment of workers for the provident fund (social security)<br>● Irregularities by employers in depositing provident fund contributions<br>Identity cards for bidi workers<br>● Employers' role in recognising and issuing identity cards for workers<br>Contractualisation in the bidi industry<br>● Contract labour system in the bidi industry and exploitation of home-based bidi makers

Trade policies concerns	Collection and sale of *tendu* leaf<br>● Indigenous communities’ ownership rights over *tendu* leaf collection<br>● Fair price to workers for sale of *tendu* leaf<br>Nationalization of *tendu* trade<br>● Potential impact on livelihoods and interests of *tendu* leaf collectors<br>Foreign direct investment in cigarette sector<br>● Potential impact on bidi trade and hence workers within the bidi sector

Health concerns

The health of tobacco workers

While many parliamentarians asked questions related to tobacco-related health harms, these concerns were about the population in general and not specifically about Indigenous communities. The reference to Indigenous communities, in such debates, was incidental as part of government responses where specific policies/programs related to Indigenous communities and/or forest areas were mentioned.

Whenever health-related concerns were brought up by parliamentarians that specifically linked tobacco and Indigenous communities, they were typically framed around Indigenous community members as workers in tobacco supply chains, especially *tendu*-leaf collectors, bidi rollers and occasionally other aspects of tobacco supply chains (e.g., as tobacco barn owners or tobacco farmer/farm worker). Bidi is a smoked tobacco product, more prevalent than cigarettes in India, wherein processed tobacco is hand-rolled using *tendu* leaf (Diospyros melanoxylon) and tied with a cotton thread.(13)

Some of the specific health concerns were related to inadequate nutrition and tuberculosis (with workers exposed to fine tobacco dust).

“…whether the Government is aware that the workers engaged in beedi making are suffering from the occupational hazard of T. B. Tuberculosis is permanently there among bidi workers. Lakhs of workers are affected by this but no medical care is provided to them…” (Oral Answers, Question 127, 1987)(14)

The government cited experts' advice suggesting no factual basis to the widely perceived notion that tuberculosis is an occupational risk/hazard for bidi workers.(14) Nonetheless, these concerns continued over time.

“…(a)whether there is high prevalence and mortality rate associated with tuberculosis among the women and bidi workers in the country…(d) the measures taken by the Government to

control TB and funds spent therefor and the success achieved as a result thereof during the said period…" *(Unstarred Question 2886, Lok Sabha, 2012)*(15)

The government responded by citing the Revised National Tuberculosis Control Program indicating that the program data do not point to higher rates of mortality from tuberculosis among (women) bidi workers and how the program universally promotes screening and access to free treatment for Tuberculosis to all including women and bidi workers.(15)

There were concerns about hunger and undernutrition among bidi workers from Indigenous communities.

"…(a) whether Government's attention has been drawn to a new item captioned "Hunger stalks tribal beedi workers appearing in the Times of India" April 13, 1993? (b) if so, whether Government have obtained a report on the conditions of tribal beedi workers in Andhra Pradesh (c) what steps are proposed to prevent starvation amongst the section of labour? (Starred Question 11, Rajya Sabha, 1993)(16)

The government responded by acknowledging that it has asked a report on this scenario from the state government of Andhra Pradesh.(16)

Health services for workers and their families

Apart from specific health issues affecting workers, parliamentarians expressed concerns related to inadequate access to healthcare services or the functioning of publicly funded health insurance schemes for the workers and their families.

Given there ought to be specific policy providing physical healthcare delivery facilities for bidi workers, parliamentarians raised concerns about presence and functioning of such facilities over time.

"….(a) whether the Ministry has received a request for construction of hospital for Beedi workers at Gursahaiganj in Farrukhubad District; (b) if so, the decision taken thereon; (c) if no request has been received, whether Union Government propose suo moto to establish a hospital for these beedi workers in view of their long outstanding hardships on account of lack of basic medical facilities…" (Starred-Supplementary Question 127*, Lok Sabha, 1987)*(14)

In this specific instance, the parliamentarian was also concerned about the delays in getting such a hospital in place despite repeated proposals. The government acknowledged the proposals for sanctioning a hospital and assured that it was under consideration while pointing out the expansion of dispensaries for bidi workers that was realised in the meantime.(14)

Such voices demanding healthcare services and/or accountability for functioning health insurance schemes for the bidi and *tendu* workers persisted over time.

"…(a) the number of tribal men and women in Orissa and Madhya Pradesh engaged to collect sal seeds, tendu leaves, Mahua and other forest and forests products…(c) whether any special

programme for the health care of these tribal workers and their families have been formulated?" (Unstarred Question 4940, Rajya Sabha, 2000)(17)

The government acknowledged that certain states (e.g., Andhra Pradesh and Madhya Pradesh) have come up with insurance for *tendu* leaf collectors and that Gujarat introduced a scheme to provide compensation to *tendu* leaf collectors for any injuries or deaths during working seasons.(17)

"...whether the Government is aware that RSBY centres in many districts are non-functional, if so, details thereof, State/ UT-wise, including Jharkhand along with the reasons therefor; and (e) whether the Government has taken note of irregularities in the above scheme being implemented in the interest of weavers in the country, if so, the details thereof and the action taken thereon along with the steps taken by the Government to increase its coverage particularly in tribal areas?" (Unstarred Question 4581, Lok Sabha, 2015)(18)

The RSBY (*Rashtriya Swasthya Bima Yojana*) referred to a national health insurance scheme initiated by the labour ministry (Government of India) that covered bidi workers as one of the 11 categories of workers apart from covering those living below the poverty line. The government clarified that there are no central government-run RSBY Centres as the scheme was being implemented by respective state governments.(18) The government further clarified that no specific irregularities were found regarding implementing RSBY for handloom workers.(18)

Labour welfare concerns

Many parliamentarians raised concerns about the realisation of labour rights and welfare measures for bidi workers asking for the accountability of government and employers in this regard.

Bidi workers' welfare fund

In 1976, the government of India enacted legislation that enabled the levy and collection of the cess on manufactured bidis towards creating a bidi workers' welfare fund(19) as well as using such fund towards the welfare of bidi workers(20) including the creation and improvements of public health and medical facilities, educational facilities for children of bidi workers, housing and recreational facilities, water supply and washing facilities and such other welfare measures.

Parliamentarians were concerned about the bidi manufacturers paying adequate duty (cess) towards the bidi worker welfare fund.

"...(a) the amount collected as cess as on March 1995 from Bidi factories situated in tribal districts of Gujarat State; and (b) the amount incurred on the welfare for Beedi Workers during the above period?" (Unstarred question 6334. Lok Sabha, 1995)(21)

"…(e) whether the Government are aware of irregularities being committed by major industrialists to escape from cess on Beedi; and (f) if so, the efforts being made by the Government in this regard?" (Unstarred Question 3101, Lok Sabha, 1995) (22)

There were concerns about how effectively such funds were allocated and utilised by governments towards workers' welfare.

"…(a) whether the Government of Uttar Pradesh propose to allocate funds for the construction of houses of beedi workers particularly, in the tribal areas during the last three years." (Unstarred Question 1086, Lok Sabha 1998)(23)

In these instances, the government assured to collect relevant information and disclose it to the upper house. The government assured that strict measures were being taken including the surprise checks of bidi companies by the Central Board of Excise and Customs to prevent cess evasion.

Provision of employee provident fund

Parliamentarians raised concerns about employers' role in enrolling and providing workers with the employee provident fund, a social security scheme allowing savings for the future wherein workers ought to get benefits on retirement, resignation, or death with a provision to also avail partial loans by workers for essential expenses. There were concerns that not all bidi workers were covered under the employees' provident fund.

"…(a) the number of the Beedi workers covered under the Employees Provident Fund Scheme in Andhra Pradesh in the tribal districts, (b) whether the amount deducted from the salaries of the Beedi workers are being properly credited to the Provident Fund Accounts; (c) whether a large number of the Beedi workers have not been covered under the Provident Fund Scheme, and (d) if so, the steps taken by the Government to check the malpractice?.." (Unstarred question 6530, Lok Sabha 1995)(24)

The government responded by providing the overall number (including but not specific to tribal districts) of bidi workers enrolled under the Employees Provident Fund (a total of 4,10,512 bidi workers as on 28 February 1995). The government assured that it was recovering the amount being deducted from workers' wages by employers and was crediting it to workers' provident fund accounts.(24)

"…(a) whether the government are aware of the complaints regarding fraudulent deposits in the provident funds of the beedi workers of Bihar and Gujarat due to which large scale resentment is prevailing among them…" and further seeking information about any actions taken by the government in this regard.(Question 6332, Lok Sabha, 1995)(25)

In this instance, the government responded that the relevant information is being collected and will be disclosed to the house.

Identity cards for bidi workers

One of the basic requirements for receiving such welfare benefits is to get recognised as a bidi worker by having an identity card issued by the employer to a bidi worker as statutorily mandated under the bidi Workers Welfare Fund Act. There were concerns about many workers not getting the identity cards.

"…(a) whether the Government have conducted any survey regarding the number of workers engaged in the Beedi Industry in certain States particularly in the tribal and backward areas of Gujarat State till September 30, 1995; (b) the number of identity card holders and non-identity card holders among them as on September, 1995, separately; (c) the efforts being made by the Government to liberate them from the harassment/oppression of contracts system…" (Unstarred Question 3101, Lok Sabha, 1995)(22)

In this instance, the government assured to collect and disclose the information to the house.

Contractualization in the bidi industry

The contractors and sub-contractors started playing a major role for bidi companies, especially from the 1970s onward as bidi-making became largely a home-based work from what used to be a factory-based work. There remain concerns related to the exploitation of workers rolling bidis at home by the contractors.

"…(c) the efforts being made by the Government to liberate them from the harassment/oppression of contracts system…" (Unstarred Question 3101, Lok Sabha, 1995)(22)

The government acknowledged the concern and conceded that given the historical developments resulting in the unique structure of the sector, it is "not feasible to abolish contract labour system" in the bidi sector.(22) However, it assured that state governments have been requested to prevent exploitation of workers by ensuring better implementation of legal measures including, the beedi and cigar workers (conditions of employment) act 1966, the minimum wages act 1948, and formation of the tripartite committees (involving government, industry, and worker representatives) to address disputes and labour welfare issues.(22)

Trade policy concerns

There were many concerns expressed by parliamentarians on how the government policies related to domestic and international trade would shape the domestic bidi industry in general (that include workers from Indigenous communities). These policies ranged from domestic decisions like taxation and nationalisation of *tendu* trade to measures like foreign direct investments in the tobacco sector. Here, we highlight concerns that specifically linked trade-

related policies with the livelihoods of workers from Indigenous communities engaged in bidi rolling and *tendu* leaf collection.

Collection and sale of *tendu* leaf

Tendu leaf, largely collected by Indigenous communities from the central Indian forests, is an important ingredient in the bidi industry. It is used to wrap bidi tobacco. With the tightening of forest conservation laws, parliamentarians raised concerns about access to these forests by Indigenous communities for collecting *tendu* leaves that formed an important forest-dependent livelihood option for them.

"...(a) which minor forest produces would be purchased by Tribal Cooperative Marketing Development Federation to remove exploitation of the tribals by middlemen during the current financial year" (Unstarred Question 572, Rajya Sabha, 1990)(26)

"....(a) whether the Common Minimum Programme of the Government vowed to give ownership right to Minor Forest produce (MFP) especially Tendu leaves to tribals; (b) if so, whether many States have changed track to give ownership right of minor forest produce except Tendu leaves; (c) if so, the reasons therefor; (d) whether the Government has taken up the matter with the Ministry of Environment & Forests to convince States to grant ownership right to Tribals of Tendu leaves..." (Unstarred Question 3321, Lok Sabha 2004)(27)

The government highlighted that while Indigenous communities residing in and around forests were already granted usufructuary rights (rights allowing persons to use a property not owned by them) for collecting minor forest produce for domestic consumption, there were no "clear-cut ownership rights" on minor forest produce. It suggested that while this matter belongs to legislative business/mandate of state governments, the central government will request states to legislate conferring such ownership rights to Indigenous communities for minor forest produce including *tendu* leaves.(27)

Nationalisation of *tendu* leaf trade

In the 1960s and 1970s, many state governments nationalised *tendu* leaf trade to enhance state revenues, prevent the exploitation of *tendu* leaf puckers and ensure supply for small- and medium-sized industries. When a parliamentarian raised concern on whether nationalisation will hurt Indigenous community members engaged in *tendu* leaf lucking, the government reiterated the protective impact of nationalisation on workers' rights.

"...(a) whether Government are aware that the main profession of Adivasis in Uttar Pradesh in Mirzapur district is Tendu leaves; (b) whether Government are aware of numerous sufferings of these Adivasis due to nationalisation of tendu leave trade; and (c) whether Government propose to de-nationalise the tendu leave trade for the benefit of the weaker sections of society i.e. Adivasis and if so. the salient features of the proposal?" (Unstarred Question 1008, Lok Sabha, 1977)(28)

The government clarified that *tendu* leaf collection was a seasonal activity (typically from April to June) only partially contributing to the livelihoods of Indigenous communities. It further denied the parliamentarian's claim about its exploitative impact and reiterated that the very intent of nationalising *tendu* leaf trade has been to safeguard the interests of *tendu* leaf collectors as they were not getting proper wages from contractors before such a move.(28) It clarified that the government was not considering any proposals to de-nationalise the trade.(28)

Parliamentarians kept raising concerns about state governments not buying *tendu* leaves from Indigenous communities and/or not remunerating them adequately for such purchases.

"....(a) whether millions of tribals in several districts of Madhya Pradesh are struggling for survival because of State Government refusal to purchase of tendu leaves from the tribals; (b) if so, the reasons therefore; and (c) the beneficiary oriented schemes introduced for providing alternate sources of incomes to the tribals displaced and deprived of collecting tendu leaves, sandalwood, etc." (Unstarred Question 1024, Lok Sabha 1992)(29)

In this instance, the government assured to collect information on this from respective state governments and disclose it to the house. It indicated that efforts are being made to promote local employment of Indigenous communities residing in and around forests as part of eco-development, nature conservation and afforestation initiatives/schemes.(29) In response to other related questions by parliamentarians, the government indicated that *tendu* leaf was considered one of the important minor forest produce to be promoted by the Tribal Co-operative Marketing Development Federation of India. (26)

Foreign direct investment in the cigarette sector

Parliamentarians also wondered if the promotion of foreign direct investment in the cigarette industry would negatively impact the domestic bidi industry and hence in turn the manual labour within the bidi industry (that includes members from Indigenous community).

"...(a) whether the Government have reviewed its move to allow 100% Foreign Direct Investment (FDI) in cigarette industry; (b) if so, the major advantages of the economy by permitting such investment; (c) the extent to which it is likely to go against the interests of Indigenous bidi industry especially cottage industry and the workers engaged therein;..." (Starred Question 148, Lok Sabha,1999)(30)

The government clarified that there was no ceiling in terms of foreign direct investment in cigarettes earlier. The government felt that cigarettes and bidis as different products and have very different market segments and hence enhanced foreign direct investments in the cigarette sector will only increase competitiveness in the cigarette sector without affecting the bidi industry or bidi workers.(30)

Despite government assurances, parliamentarians expressed concerns about the potential exploitation of Indigenous community members in tobacco supply chains, demanding policy

measures from the government to safeguard workers' interests. Such concerns were about *tendu* leaf collectors, bidi workers as well as a minority of people who owned tobacco barns (facilities to cure tobacco leaves).

"...(a) whether it is fact that ITDA in Andhra Pradesh has disbursed funds for tobacco barn construction in tribal areas of West and East Godavari districts in 1990, 1991 and 1992 (b) The details of funds distributed in the form of loans and in the form of subsidy to tribal tobacco farmers in the West Godavari district (c) Whether any monitoring has been done to ensure that the tobacco barns said to be owned by the tribals have not been taken over by the non-tribals and (d) in how many instances have been taken cases where such barns were constructed only with the view to draw subsidy?.." (Unstarred Question 4234, Rajya Sabha, 1994)(31)

"Whether Tendu leaf pluckers, most of whom are tribals in Madhya Pradesh, Chhattisgarh and Orissa, have demanded their share from the commercial exploitation of Tendu leaf and bamboos;..." (Unstarred Question 1886, Lok Sabha, 2002)(32)

"...(a) which minor forest produces would be purchased by Tribal Cooperative Marketing Development Federation to remove exploitation of the tribals by middlemen during the current financial year" (Unstarred Question 572, Rajya Sabha, 1990)(26)

Discussion

In this paper, through analysis of parliamentary questions over time (1952-2022), we describe major concerns that parliamentarians have expressed about tobacco in relation to Indigenous communities. We find that, overall, such concerns linking tobacco and Indigenous communities constitute a very small part (284 questions in the 70-year period from 1952 to 2022) of the total tobacco-related questions by parliamentarians (1315 questions only in the 20-year period from 1999 to 2019)(12). Furthermore, a very tiny proportion of parliamentarians expressing concerns linking tobacco and Indigenous communities came from Indigenous communities. Their concerns were largely about marginal workers from Indigenous communities in tobacco supply chains: about their health issues and access to healthcare services, their exploitation by employers and potential threats to their interests and already precarious livelihoods. We now discuss some of these concerns by parliamentarians in the context of what we know from empirical studies on these issues as well as their implications for tobacco control and public health measures among Indigenous communities.

Invisible and precarious workforce within tobacco supply chains

Parliamentarians' concerns bring the spotlight on members of Indigenous communities that form a precarious workforce within tobacco supply chains (especially as bidi makers, *tendu* leaf collectors and small-holder tobacco farmers), something that is hardly discussed in tobacco control discourse in India. Many studies corroborate concerns raised by parliamentarians about occupational risks and health hazards associated with such work as well as economic exploitation and poor state of labour rights/welfare of these workers despite the presence of

specific regulatory provisions protecting these workers.(33–35) Studies highlight that members of Indigenous communities often take up tobacco-related work in the absence of alternative livelihood opportunities including lack of, or poor implementation of, public schemes assuring employment guarantees.(36,37)

While the WHO Framework Convention on Tobacco Control (a treaty signed and fully ratified by the Government of India) recognises the importance of promoting economically viable and healthy alternatives to tobacco-dependent livelihoods, there has been very limited progress on this front.(38,39) This study, along with corroborating evidence discussed earlier, implies the need for a just transition wherein the tobacco supply reduction measures shall go hand in hand with measures that ensure fair remuneration and work conditions for the marginal workers in tobacco supply chains and that promote access to economically viable and acceptable livelihoods alternatives.

Health concerns are largely limited to the 'worker identity'

What is intriguing is that parliamentarians' concerns about health issues including access to healthcare services are primarily about workers from Indigenous communities engaged in tobacco supply chains. We did not come across any concerns about tobacco use and its health harms among Indigenous communities. This is despite the fact that parliamentarians have been increasingly concerned about the health harms of tobacco in general and tobacco use has remained disproportionately high among many Indigenous communities, who also experience poorer access to healthcare services/support. This could possibly imply that parliamentarians are yet to recognise and prioritise the challenge of substance use (especially tobacco use in this case) in addressing the health of Indigenous communities. This could also reflect a poor representation of Indigenous communities in the Indian parliament. The fact that the Indigenous communities have experienced the least decline in tobacco use over the last few decades, it is important to develop a special focus on Indigenous communities within tobacco control efforts, making them specifically relevant, acceptable, and informed by voices from Indigenous communities. In this respect, the recent efforts in this direction including the first-of-its-kind national conference on tobacco use among tribal communities by the All India Institute of Medical Sciences (Deoghar)(40) as well as our efforts at understanding historical and socio-cultural pathways of substance use among Indigenous communities at the Centre for Training, Research and Innovations in Tribal Health(7) are relevant. However, there is a long way to go, and the agenda needs to be about developing acceptable and effective interventions beyond simply expanding the existing approaches to tribal areas.

Tobacco's impact on forests and environment: a missing link?

Like the health harms of tobacco consumption, the negative impact of tobacco on forests and the environment seems to be another, more indirect, link to the health and wellbeing of Indigenous communities. The forests remain central to the survival and sustenance of forest-dwelling Indigenous communities. Tobacco, throughout its lifecycle, is known to have a negative impact on the environment and forests.(41) In India, we have only started to map the magnitude of this problem: be it deforestation from *tendu* harvesting-related forest fires(42)

and illegal logging of fuelwood for tobacco curing(43) or the environmental pollution resulting from tobacco production, consumption, and hazardous tobacco product waste.(44)

Public health research and advocacy community need to expand their remit to engage with complex and multiple ways in which tobacco use and the tobacco industry are impacting Indigenous communities to make any positive difference.

Limitation

There are limitations of our study. The digital search catalogues of the lower and the upper houses of the Indian parliament that we used to source the transcripts are well-optimised for specific searches as they do not recognise Boolean operators. There is no specific guidance provided to optimise their use. This could have limited the number of relevant transcripts we could source despite the elaborate searches we ran. Apart from the low representation (in terms of absolute numbers) of the Indigenous communities in the Indian parliament, the study of the parliamentary proceedings over 30 years (1980-2009) by Ayyangar and Jacob(45) reveals that parliamentarians from scheduled tribe category, as well as women parliamentarians, participate less actively (asking a lesser number of questions on an average) in the Question Hour compared to men and parliamentarians from upper castes. This is an important limitation as the voices of parliamentarians from Indigenous communities remain limited and so are the voices of women (most bidi makers and many of the *tendu* leaf collectors are women). They also highlight that participation of parliamentarians from the northeastern states of India remains limited – these are the states with a greater proportion of Indigenous communities as well as a high prevalence of tobacco use.(45) Finally, the very procedure for and the nature of proceedings during the Question Hour are such that puts a limit to the length and candid nature of transactions among parliamentarians.

Conclusions

Overall, the representation of Indigenous communities in the Indian parliament remains very limited. Parliamentarians, over time, expressed varied concerns linking tobacco and Indigenous communities in India. The major concerns included (1) occupational and other health hazards as well as inadequate access to healthcare services faced by members from Indigenous communities working as bidi makers and *tendu* leaf collectors; (2) unfair wages, exploitative work conditions and inadequate social security to these workers; and (3) perceived negative impact on tobacco-linked livelihoods resulting from trade and investment related policies in the tobacco sector. Parliamentarians did not raise issues related to high tobacco use and tobacco-related harms among Indigenous communities as well as the negative impact of tobacco on forests that are central to the lives of Indigenous communities.

Acknowledgement

We would like to acknowledge Soniya SM, pursuing her public health degree at the Karnataka State Rural Development and Panchayat Raj University, for her support in data management during her three-month internship with us.

References

1. Kaur J, Rinkoo AV, Richardson S. Update on numbers of tobacco-attributable deaths by country in the South-East Asia region: implications for policy. Tob Control. 2024;0:1–4. doi:10.1136/tc-2024-058599
2. Ministry of Health and Family Welfare & Ministry of Tribal Affairs. Government of India. Report of the expert committee on tribal health. Tribal health in India: bridging the gap and a road map for the future. 2018. Available from: https://tribalhealthreport.in/full-report/
3. DBT/Wellcome Trust India Alliance. 11th India Alliance annual conclave 2023: abstract book. Hyderabad: DBT/Wellcome Trust India Alliance; 2023. [cited 2025 April 10] Available from: https://dbtindiaalliancenew.s3.ap-south-1.amazonaws.com/jsodmzclnfwpaha1725602975586/IAAC-2023-Abstract-Book-updated-12-July.pdf
4. Ministry of Health & Family Welfare Government of India. Tobacco control laws and related laws in India. National Tobacco Control Program. 2024 [cited 2025 April 10]. Available from: https://ntcp.mohfw.gov.in/acts_rules_regulations
5. Ministry of Health & Family Welfare Government of India. GATS 2: Global adult tobacco survey, factsheet India 2016-17. [cited 2025 April 25] Available from: https://www.mohfw.gov.in/sites/default/files/GATS-2 FactSheet.pdf
6. Hebbar PB, Bhojani U, Schayck O Van, Babu G, Dsouza V. Shifting the gaze on implementation : examining the association between the implementation of tobacco control laws and prevalence of tobacco using data from a nationally representative survey. BMC Public Health. 2023;1–9. Available from: https://doi.org/10.1186/s12889-023-16780-8
7. Institute of Public Health Bengaluru. Centre for Training, Research, and Innovation in Tribal Health (CTRITH). 2025. [cited 2025 April 10] Available from: https://iphindia.org/centre-for-training-research-and-innovation-in-tribal-health-ctrith/
8. General Information. Lok Sabha (House of the People). 2025. [cited 2025 April 10] Available from: https://sansad.in/ls/questions/questions
9. Introduction: Question Hour. Rajya Sabha (Council of States). 2025. [cited 2025 April 10] Available from: https://sansad.in/rs/questions/general-information
10. Das SS. Traditional healing among the Tai Ahoms of Assam: changing dynamics and its interaction with the public health system. PhD Thesis. Jawaharlal Nehru University, New Delhi; 2024.
11. Das SS. Witch-Hunting from a public health perspective: narratives from Assam. MPhil Thesis. Jawaharlal Nehru University, New Delhi; 2018.
12. Varma A, Chilgod L, Bhojani U. Diverse and competing interests around tobacco: Qualitative analysis of two decades of parliamentary questions in India. BMJ Glob Heal. 2021;6(4):1–10. Available from: https://gh.bmj.com/content/6/4/e004093
13. Gupta PC, Asma S. Bidi smoking and public health. Ministry of Health Family Welfare. New Delhi; 2008. Available from: https://ntcp.mohfw.gov.in/assets/document/Monograph on Bidi Smoking and Public Health.pdf
14. Parliament of India. Oral answers, Question 127. 1987. p. 20. [cited 2025 April 10] Available from: https://eparlib.nic.in/bitstream/123456789/878354/1/08_IX_16-11-1987_p13_p15_s127.pdf
15. Parliament of India. Question No: 2886. 2012. [cited 2025 April 10] Available from: https://eparlib.nic.in/bitstream/123456789/616866/1/118419.pdf
16. Parliament of India (Rajya Sabha). Question 11. 1993. p. 39–40.

17. Parliament of India (Rajya Sabha). Question 4940. 2000. p. 87.
18. Parliament of India (Lok Sabha). Question 4581. 2016. [cited 2025 April 10] Available from: https://eparlib.nic.in/bitstream/123456789/688010/1/40331.pdf
19. The Beedi Workers Welfare Fund Act, 1976. [cited 2025 April 10] Available from: https://labour.gov.in/sites/default/files/thebeediworkerswelfarefundact1976.pdf
20. The Beedi and Cigar Workers (Conditions of Employment) Act, 1966. Government of India. [cited 2025 April 10] Available from: https://www.indiacode.nic.in/handle/123456789/1675?sam_handle=123456789/1362
21. Parliament of India (Lok Sabha). Question 6334. 1995. p. 43. [cited 2025 April 10] Available from: https://eparlib.nic.in/bitstream/123456789/2392213/1/10_XIII_19-05-1995_p26_p26_u6332.pdf
22. Parliament of India (Lok Sabha). Question 3101. 1995. p. 485–6. [cited 2025 April 10] Available from: https://eparlib.nic.in/bitstream/123456789/2402879/1/10_XV_18-12-1995_p247_p247_u3101.pdf
23. Parliament of India (Lok Sabha). Question 1086. p. 305. [cited 2025 April 10] Available from: https://eparlib.nic.in/bitstream/123456789/2963091/1/US_1086_02-06-1998_1.pdf
24. Parliament of India (Lok Sabha). Question 6530. 1995. p. 174. [cited 2025 April 10] Available from: https://eparlib.nic.in/bitstream/123456789/2392411/1/10_XIII_19-05-1995_p91_p91_u6530.pdf
25. Parliament of India (Lok Sabha). Question 6332. 1995. p. 43. [cited 2025 April 10] Available from: https://eparlib.nic.in/bitstream/123456789/2392213/1/10_XIII_19-05-1995_p26_p26_u6332.pdf
26. Parliament of India (Rajya Sabha). Question 572. 1990.
27. Parliament of India (Lok Sabha). Question 3321. 2004. [cited 2025 April 10] Available from: https://eparlib.nic.in/bitstream/123456789/572298/1/7431.pdf
28. Parliament of India (Lok Sabha). Question 1008. 1977. p. 91.
29. Parliament of India (Lok Sabha). Question 1024. 1992. p. 559. [cited 2025 April 10] Available from: https://eparlib.nic.in/bitstream/123456789/2349633/1/10_IV_14-07-1992_p287_p287_u1024.pdf
30. Parliament of India (Lok Sabha). Question 148. 1999. p. 34–5. [cited 2025 Aprill 10] Available from: https://eparlib.nic.in/bitstream/123456789/793655/1/12_IV_05031999_p21_p22_s148.pdf
31. Parliament of India (Rajya Sabha). Question 4324. 1994.
32. Parliament of India (Lok Sabha). Question 1886. 2002. [cited 2025 April 10] Available from: https://eparlib.nic.in/bitstream/123456789/433857/1/48211.pdf
33. Government of India. Evaluation study on the implementation of Minimum Wages Act, 1948 in beedi making industry in Madhya Pradesh. Chandigarh; 2015. [cited 2025 April 10] Available from: https://labourbureau.gov.in/uploads/pdf/Report_on_Beddi_Industry_2015.pdf
34. Status of tendu leaf pluckers in Orissa: a study of their socio-economic conditions with special reference to children and the system of bonded labour. Delhi; 2006. [cited 2025 April 10] Available from: http://hdsindia.org/wp-content/uploads/2018/06/NHRC-Tendu-Leaf-Pluckers-Report.pdf
35. Rathore A, P. Ugargol A. Beedi rolling and the health hazards associated with occupational exposure: a situational review from Karnataka, India. RGUHS Natl J Public Heal. 2018;4(1). Available from: https://rnjph.journalgrid.com/view/article/rnjph/653
36. Sabar B, Nayak N, Achoth L. Tendu leaves collection in India: livelihoods, rights and

challenges for alternative to tobacco - evidences from five Indian states. J Gov Public Policy. 2016;6(1):106-123.

37. Russell A, Chandra P, Robson M, Narayanan P, Joseph S, Mukherjee P, et al. Implementing FCTC Article 17 Through Participatory Research With Bidi Workers in Tamil Nadu, India. Nicotine Tob Res. 2022;24(11):1714–9. Availabe from: https://doi.org/10.1093/ntr/ntac075
38. World Health Organization. WHO Framework Convention on Tobacco Control. Geneva; 2003. Available from: https://fctc.who.int/resources/publications/i/item/9241591013
39. Chung-Hall J, Craig L, Gravely S, Sansone N, Fong GT. Impact of the WHO FCTC over the first decade: A global evidence review prepared for the Impact Assessment Expert Group. Tob Control. 2019;28:S119–28. Available from: https://tobaccocontrol.bmj.com/content/28/Suppl_2/s119
40. All India Institute of Medical Sciences (Deoghar). 1st National Conference on "Tobacco Use among Tribal Communities" 20-21 July 2024. [cited 2025 April 10] Deoghar; 2024. Available from: https://www.aiimsdeoghar.edu.in/Content/resources/document/notice/national_conference_tutc_2024_19062024_050827183.pdf
41. World Health Organization. Tobacco and its environmental impact: an overview. 2017 [cited 2025 April 10] Available from: http://dx.doi.org/10.1016/j.ccm.2013.09.011
42. Singh M, Sah P, Kumar P. Forest fires and tendu leaf collection in India: an evidence-based analysis. New Delhi; 2023. [cited 2025 April 10] Available from: https://iforest.global/research/report_forest-fires-and-tendu-leaf-collection-in-india-an-evidence-based-analysis-request-download/
43. Nayak NS. Tobacco Curing and Fuel Efficiency in Karnataka, India. Kathmandu; 2013. Available from: http://www.sandeeonline.org/uploads/documents/publication/1007_PUB_Working_Paper_77_Nayantara.pdf
44. All India Institute of Medical Sciences Jodhpur, Indian Council of Medical Research - National Institute of Cancer Prevention and Research, The International Union against Tuberculosis and Lung Diseases. The Environmental Burden of Tobacco Products Wastes in India: tobacco use makes you ugly, your habitate uglier. 2022. [cited 2025 April 10] Available from: https://nicpr.org/wp-content/uploads/2023/02/tobacco_waste_report.pdf
45. Ayyangar S, Jacob S. Studying the indian legislature: What does question hour reveal? Stud Indian Polit. 2014;2(1):1–19. doi: 10.1177/2321023014526023

Figure 2

Click here to access/download;Figure;Fig2.tiff

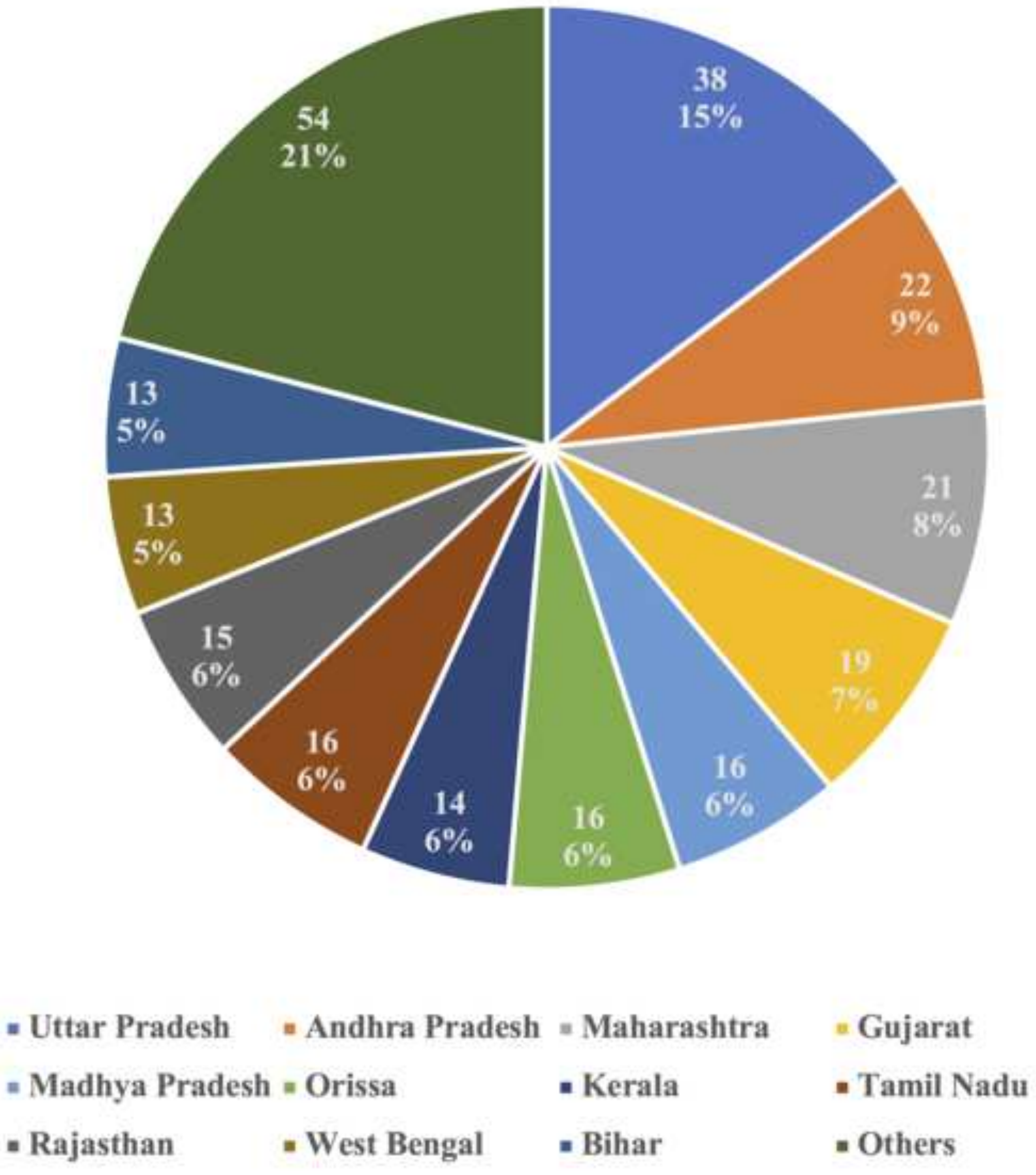

Figure 3

Click here to access/download;Figure;Fig3.tiff

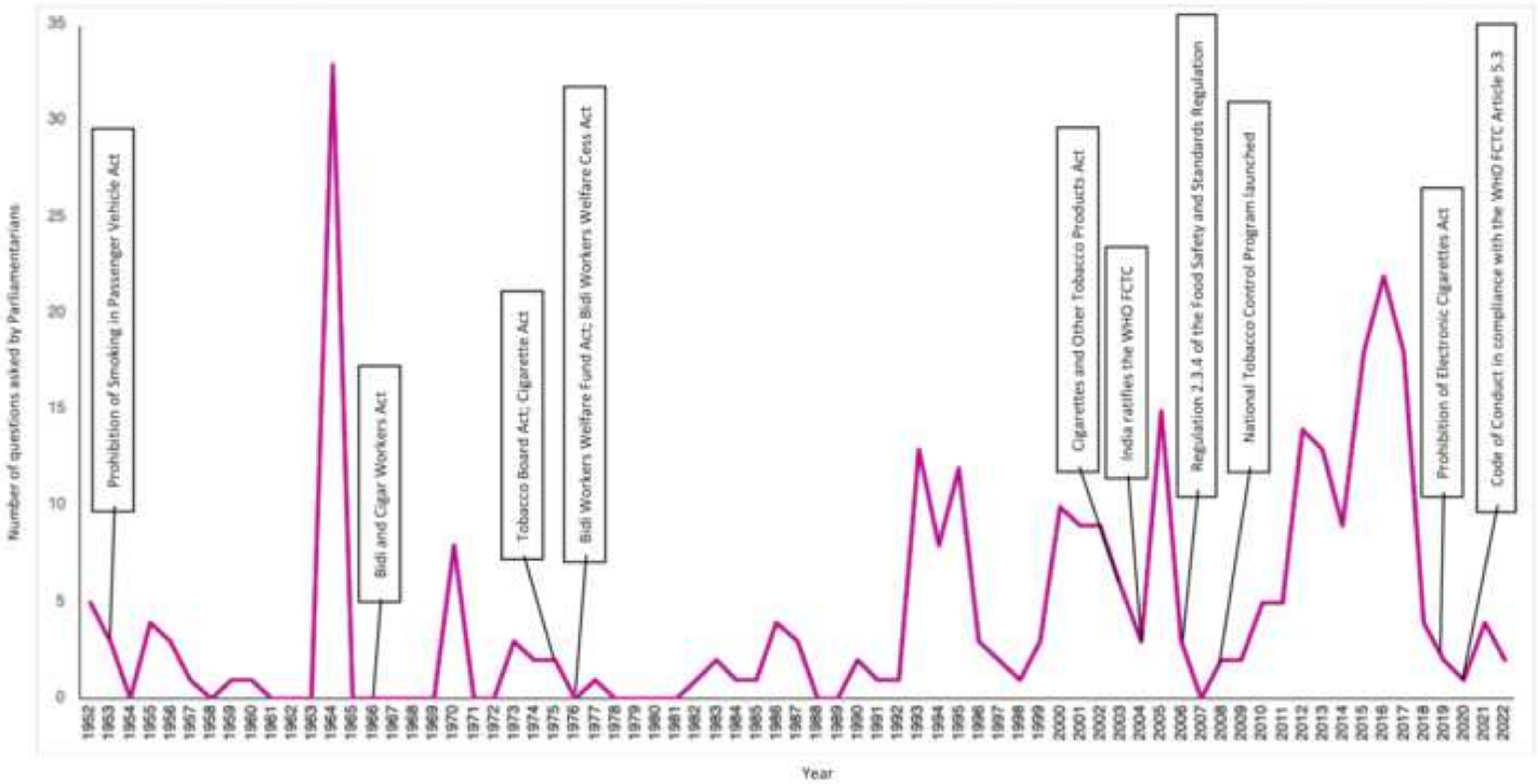

Figure 1

Click here to access/download;Figure;Fig1.tiff

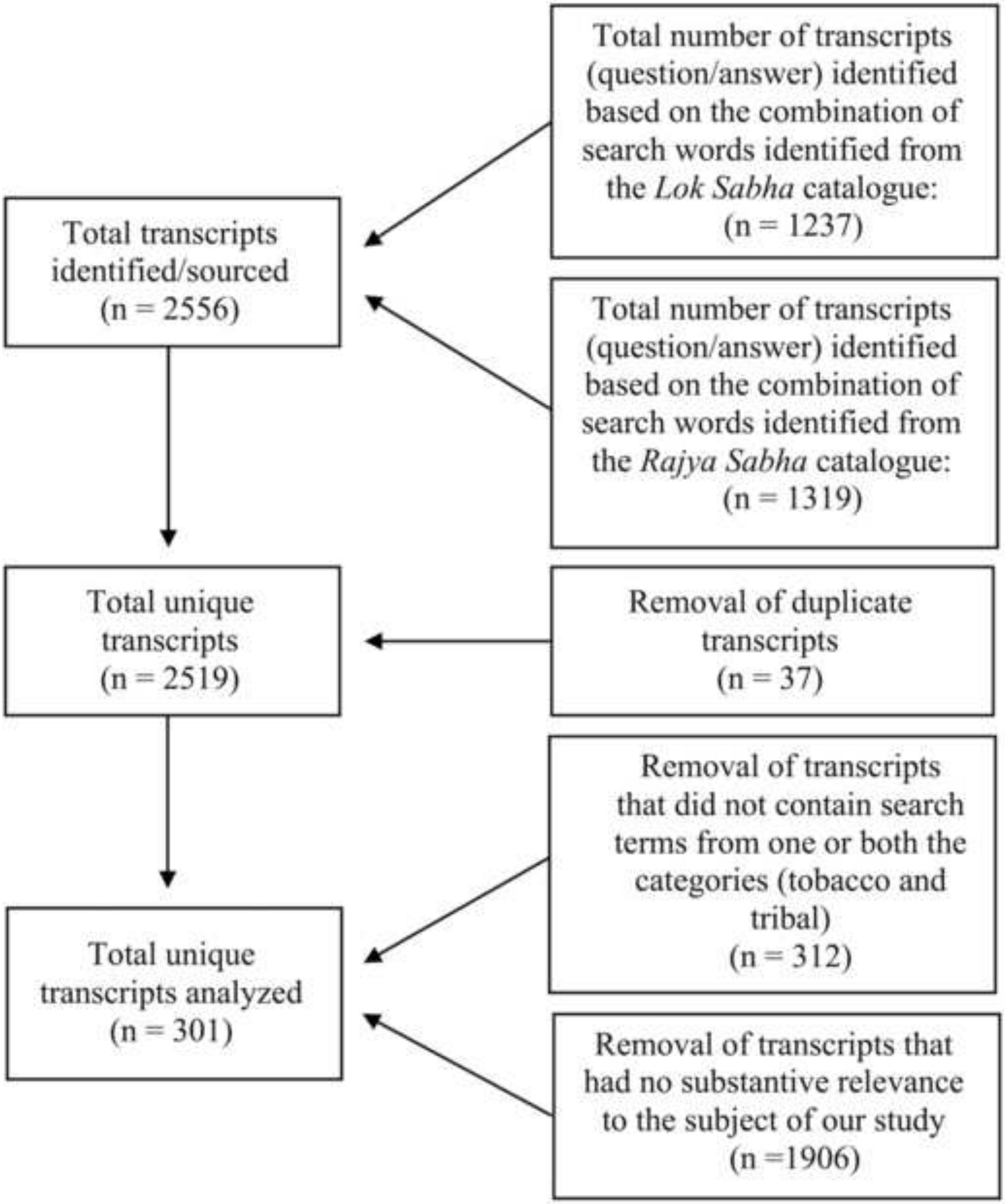

~~Parliamentarians~~Policymakers’ concerns linking tobacco and I~~i~~ndigenous communities in India: a qualitative analysis of parliamentary questions (1952-2022)

Shilpi Sikha Das[1,2#], Upendra Bhojani[1,2#*], Prashanth NS[1]~~, Upendra Bhojani[1,2*]~~

Formatted: Not Superscript/ Subscript

[1]Centre of Adivasi Health, Institute of Public Health, Bengaluru, Karnataka, India

[2]Centre for Commercial Determinants of Health, Institute of Public Health, Bengaluru, Karnataka, India

[#]Contributed equally

Formatted: Superscript

[*]Corresponding author

Email: upendra@iphindia.org

Abstract

Tobacco use and associated disease burden remains high among ~~indigenous~~ Indigenous communities in India. Despite an overall decline in tobacco consumption over the last few decades, the social disparities in tobacco use have widened with ~~indigenous~~ Indigenous communities experiencing the least decline. Existing tobacco control policies lack specific considerations for ~~indigenous~~ Indigenous communities. Hence, as part of a broader research initiative focusing on the health of ~~indigenous~~ Indigenous communities, we explored how parliamentarians in India have framed their concerns about tobacco in reference to ~~indigenous~~ Indigenous communities over time.

We sourced digital transcripts of exchanges (parliamentarians asking questions and ministers responding to them) as part of the Question Hour sessions in the lower- and upper- houses of the Indian parliament between 1952 and 2022. We used thematic content analysis for 301 transcripts that linked tobacco and ~~indigenous~~ Indigenous communities. Overall representation of ~~indigenous~~ Indigenous communities in the Indian parliament remains very limited. The major concerns expressed by parliamentarians included (1) occupational health hazards and inadequate access to healthcare services faced by members from ~~indigenous~~ Indigenous communities working as bidi makers and tendu leaf collectors;(2) unfair wages and exploitative work conditions for these workers; and (3) perceived negative impact on tobacco-linked livelihoods resulting from trade and investment related policies in the tobacco sector. Parliamentarians did not raise issues related to high tobacco use and tobacco-related harms among ~~indigenous~~ Indigenous communities in general, nor did they talk about the negative impact of tobacco on forests that are central to the lives of ~~indigenous~~ Indigenous communities.

Public health research and advocacy efforts need to acknowledge the complex and multiple intertwining links between tobacco (industry) and ~~indigenous~~ Indigenous communities.

There is a need to sensitise policymakers on the health and environmental impacts of tobacco while addressing the prevailing exploitation of workers from ~~indigenous~~ Indigenous communities in precarious tobacco supply chains and providing viable alternative livelihoods.

Keywords

~~Indigenous~~ Indigenous communities; Adivasis; India; tobacco; health policy; parliament; tribal health

Introduction

The widespread tobacco consumption in India poses a huge public health challenge. Tobacco consumption accounts for about 1.94 million adult deaths annually in India apart from a significant morbidity burden. ~~(~~(1) In India, tobacco consumption varies widely across social groups. The report of the Expert Committee on Tribal Health set up by the Government of India highlighted that while tobacco use among ~~indigenous~~ Indigenous communities varied across states, in general, it remained high among ~~indigenous~~ Indigenous communities.(2) It indicated that as per the National Nutrition Monitoring Bureau survey (2008-2009) covering nine Indian states, 35.9% of men and 6.3% of women in ~~indigenous~~ Indigenous communities (categorised as 'Scheduled Tribes' as per the Indian constitution) smoked tobacco.(2) Furthermore, 37.7% of men and 23.1% of women from ~~indigenous~~ Indigenous communities chewed tobacco.(2) The report indicated that the true prevalence of tobacco use among ~~indigenous~~ Indigenous communities might be higher as the nationally representative survey (National Family Health Survey) in 2005-2006 indicated the overall prevalence of tobacco use among men from Scheduled Tribe category to be 71.7%, much higher compared to men not belonging to Scheduled Tribes (56.3%).(2) A recent analysis of a nationally representative study (Longitudinal Ageing Study in India) also pointed high burden of tobacco use among Indigenous communities. It estimated that in the year 2017-2018, the prevalence of tobacco use among Scheduled Tribes (aged 45 years and above) was 46%.(3) ~~Our earlier work (data unpublished) analysing the comparable rounds of the National Sample Survey revealed that such disparity in tobacco use existed in 1990 with people belonging to the Scheduled Tribe category experiencing the highest prevalence of tobacco use. (3) While the overall prevalence of tobacco use declined over the 1990-2012 period that we studied, the disparity across social groups widened with people in the Scheduled Tribe category experiencing the least decline in tobacco consumption. ((3)~~ This implies that public health policies/programs and the broader social determinants favourable to the reduction in tobacco use in India have not equitably benefited all the social groups in India.

This concern about high levels of tobacco use among indigenous Indigenous communities is not limited to India. Studies have found that in several countries, especially in Australia, New Zealand, United States of America and Canada, the prevalence of commercial tobacco use among Indigenous Indigenous communities has been much higher compared to non-Indigenous Indigenous communities.(4) Tobacco industry has been recognised as one of the important commercial determinants affecting health and wellbeing of Indigenous Indigenous communities.(5) Many countries have recognised this and have developed specific strategies to reduce such disparities.(6) For example, Australia, in its National Drug Strategy (2017-2026) identifies Aboriginal and Torres Strait Islanders people as priority groups with a specific sub-strategy for these communities indicating need for careful, contextual and participative interventions.(7) Canada's Tobacco Strategy prioritises Indigenous Indigenous communities ensuring specific funding to First Nations, Inuits, and Metis Nation people enabling development of their own plans to address commercial tobacco use.(8)

India is home to 7305 ethnic groups that are officially notified as 'Scheduled Tribes'. According to the Census of India, (2011), -104.5 million people belonged to the 'Scheduled Tribe' category constituting 8.6 percent of the total population in India. -(910) Indigenous Indigenous communities in India are highly diverse in terms of culture, language, social organization, and their \-habitat. In general, Indigenous Indigenous communities have low rates of literacy, access to health infrastructure and poor nutrition and health indicators.(9) There are constitutional safeguards to protect and promote rights of Scheduled Tribes including reservations in higher education and election seats.(10)\

In India, TThere have been several policy initiatives by the Government of India for reducing tobacco use (11)4) and these initiatives seem to be making some positive impact.(12, 135,6). However, there is no specific focus or considerations for Indigenous Indigenous communities within these initiatives. As part of the Centre for Training, Research and Innovation in Tribal Health, a multi-institutional research initiative, we have been researching substance use (among other health issues) and haemoglobinopathies among indigenous Indigenous communities.(14)20) As part of this broader research agenda, we are conducting research on various aspects of substance use among indigenous Indigenous communities: –from exploring sociocultural pathways of substance use among local communities to mapping policy discourse related to substance use among indigenous Indigenous communities over time. As parliamentarians play a crucial role in shaping policy discourse and policymaking, including for tobacco control, we aimed to study how parliamentarians in India framed their concerns related to tobacco and indigenous Indigenous communities in India over time. Our earlier analysis of pParliamentarians'' concerns related to tobacco in general - not specific to Indigenous Indigenous communities - indicated that pParliamentarians were increasingly concerned about health harms of tobacco and the need for tobacco control measures amidst continuing concerns about tobacco-linked revenues and livelihoods.(15) We presumedanticipated that pParliamentarians will be interested in issues affecting Indigenous Indigenous communities given they are considered special groups who suffered historical injustice and who have

Formatted: Font: 12 pt

Formatted: Font: 12 pt

Formatted: Font: 12 pt

Formatted: Font: 12 pt

Formatted: Font: 12 pt

Formatted: Font: 12 pt

Formatted: Font: 12 pt

Formatted: Font: 12 pt, Font color: Text 1

Formatted: Font: 12 pt

Formatted: Font: 12 pt

Formatted: Font: 12 pt

Formatted: Font: 12 pt

Formatted: Justified, Right: 0.2"

constitutional safeguards.(10) In fact, there is a ~~seperate~~separate Ministry of Tribal Affairs within the Government of India. Hence, we aimed to better understand how ~~p~~Parliamentarians framed their concerns linking tobacco and ~~Indigenous~~ Indigenous communities over time. Such understanding will inform how tobacco has been perceived and/or problematised concerning ~~indigenous~~ Indigenous communities in the Indian parliament with implications on tobacco control advocacy efforts with policymakers in India.

Methods

Study design and setting

We used a qualitative approach~~study~~ wherein we analysed the digital transcripts of questions and answers that referred to tobacco and ~~indigenous~~ Indigenous communities in India as part of the parliamentary proceedings in both the houses of Indian parliament. India is a union of states with a representative parliamentary democracy and a bicameral legislature. We chose to source the digital transcripts of questions and answers happening as part of the Question Hour of the lower (*Lok Sabha*)(17) and the upper (*Rajya Sabha*)(18) houses of the Indian parliament. The Question Hour is a platform wherein concerned ministers respond to the prior-submitted questions by any members of parliament. These ~~((21,22)~~ questions can cover any aspect~~s~~ of government functioning and administration as well as raising concerns of their electorates. Hence, the idea is to promote government transparency and accountability to the electorate.(17, 18~~21,22~~) Our study focused on analysing questions/answers starting from the year 1952 (the year Question Hour was mooted, and digital transcripts are available from the web portals of the parliament) till the end of the year 2022.

Data collection

We searched the publicly available digital catalogues of parliamentary questions from both houses; Lok Sabha (Lower House)(19) ~~https://sansad.in/ls/questions/questions-and-answers~~ and Rajya Sabha (Upper House)(20) ~~https://sansad.in/rs/questions/questions-and-answers~~ separately using a combination of search terms ‘tobacco’ and ‘tribal’ within the full text of the transcripts without any search filters. We used synonyms/substitutes of these terms (for tobacco: *bidi, beedi, biri, tendu, gutka, cigarette, hookah, hukka, kharra, gul, mishri, mawa, gudakhu, nastaar, khaini, chillum,* cigar, *and cheroot*; for tribal: *adivasi, adiwasi, and*

Formatted: Justified

indigenous) in all possible combinations. A total of 2556 transcripts were sourced with the oldest dating May 29, 1952, and the most recent dating July 26, 2022. Such a search strategy was arrived at after trying various searches to better understand how these search catalogues work (e.g., they do not recognise Boolean operators). The search results were exported into MS Excel spreadsheet and duplicates were removed using the relevant function. The first author (SSD) read all the transcripts in full and further excluded those that did not contain at least one of the two categories of search terms (tobacco and tribal) or had these terms occurring incidentally (often in answers) while the transcript had no substantive relevance to the subject of our study. Fig 1 provides details about the sourcing and screening outcomes. The first author ~~reviewed all the questions and strictly excluded those that did not meet the criteria. She~~ then discussed the shortlisted questions~~,~~ as well as potential exclusion that lacked clarity with the second author (UB) and made a decision about inclusion/exclusion based on consensus. In case of disagreement, the matter was referred to the third author (PNS), who discussed with the first two authors and made a final decision. ~~The decision about the exclusion of transcripts due to non-relevance to the study subject was taken through discussion and consensus between the first (SSD) and the last (UB) authors.~~ We included a total of ~~analysed~~ 301 transcripts for analysis in this study. ~~this study.~~

Fig 1 Selection of transcripts of parliamentary questions for analysis (1952-2022)

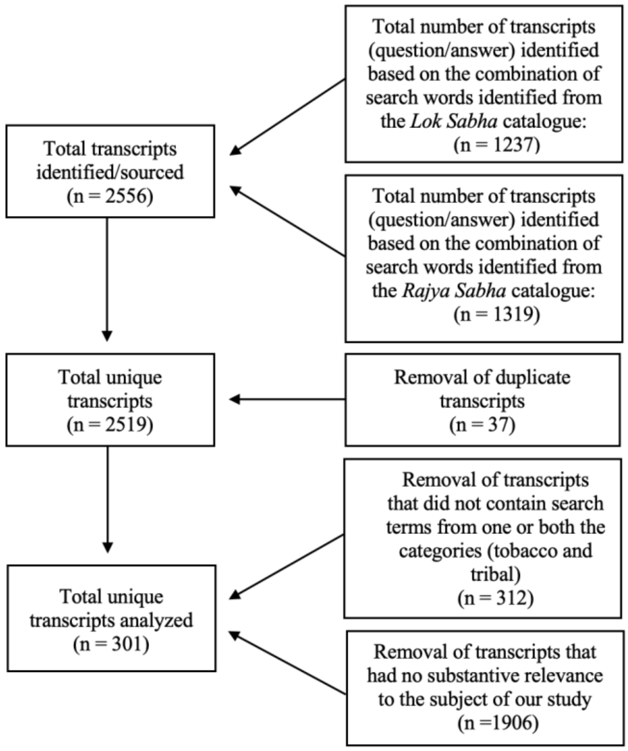

Data management and analysis

The transcripts were typically a few pages long. An MS Excel spreadsheet was used to create a dataset wherein each row represented a unique question (and answer) asked in the parliament. Various attributes related to that question were recorded (e.g., date when asked, parliamentarian/s asking the question, the parliamentary house, the ministry which was asked that question). Some of the questions were asked by more than one parliamentarian, and some of the parliamentarians had asked more than one question related to tobacco and ~~indigenous~~ Indigenous communities. Hence, another MS Excel spreadsheet was used to create a dataset wherein each raw represented a unique parliamentarian. Various attributes of that parliamentarian were recorded (e.g., gender, political party affiliation, constituency, and the state that the parliamentarian represented).

We used thematic content analysis(21) using an interpretive lens to analyse transcripts ~~(23,24)~~. As this was an exploratory work with no precedent of similar inquiry (linking tobacco and ~~indigenous~~ Indigenous communities), the first author (SSD) used inductive coding of transcripts, guided by the overall question of 'how parliamentarians were framing their tobacco-related concerns in relation to the ~~indigenous~~ Indigenous communities?' rather than using any specific theoretical framework. She periodically discussed the coding pattern and the emerging themes she articulated based on coding with the second~~last~~ author (UB). Both these authors had formal training in qualitative research. The first author has worked with ~~indigenous~~ Indigenous communities for her doctoral and post-doctoral work.(225,236) The last author has been researching tobacco control for about two decades including published work using parliamentary questions. –(1525) He has been collaborating with scholars working with ~~indigenous~~ Indigenous communities including some of the members from the ~~indigenous~~ Indigenous community. Both authors, through periodic discussions and iterations, agreed on the overall themes and explored the interrelations across themes. These findings are presented using themes and specific quotes from the data. At some point, both datasets were combined through unique IDs given to transcripts to describe the data using attributes of the parliamentarians (presented in charts/tabular format).

Formatted: Justified

Results

About the parliamentarians

A total of 257 parliamentarians asked 301 questions over the 70-year period (1952-2022) linking tobacco and ~~indigenous~~ Indigenous communities. Most of them were in the lower house of parliament (76.7%) and were men (89.5%). Only 9.5% of these parliamentarians belonged to the Scheduled Tribe category. Over 60% of them belonged to the two major national political parties: Congress accounting for 36.2% and Bharatiya Janata Party accounting for 24.1% of these parliamentarians. The remaining 40% of parliamentarians belonged to 31 different political parties including independent and nominated candidates. These parliamentarians represented constituencies across 28 states (including union territories and erstwhile

jurisdictions) with Uttar Pradesh (15%), Andhra Pradesh (9%) and Maharashtra (8%) accounting for a third of these parliamentarians. Table 1 and Fig 2 provide details about select attributes of these parliamentarians. Nearly three fourth (73.8%) of the total questions (analysed in this study) were asked in post 1990 period that typically saw frequent tobacco control reforms. In general, questions spiked preceding major tobacco-related reforms (see Fig 3).

Formatted: Justified

Table 1 Parliamentarians asking questions about tobacco and ~~indigenous~~ Indigenous communities (1952-2022)

Political party	Total	Parliament House		Gender		Social Group	
		Lok Sabha (Lower House)	*Rajya Sabha* (Upper House)	Men	Women	Scheduled Tribe	Others
Congress	36.2% (93)	36.5% (72)	35% (21)	37% (85)	29.6 % (8)	28% (7)	37.1% (86)
Bharatiya Janata Party	24.1% (62)	25.9% (51)	18.3% (11)	23% (53)	33.3 % (9)	32% (8)	23.3% (54)
Independent and nominated candidates	3.9% (10)	3% (6)	6.7% (4)	3.9% (9)	3.7 % (1)	0% (0)	4.3% (10)
All India Anna Dravida Munnetra Kazhagam	3.5% (9)	4.1% (8)	1.7% (1)	3.5% (8)	3.7% (1)	4% (1)	3.4% (8)
Communist Party of India (Marxist)	3.5% (9)	3% (6)	5% (3)	3.5% (8)	3.7% (1)	4% (1)	3.4% (8)
Telugu Desam Party	3.1% (8)	2% (4)	6.7% (4)	2.6% (6)	7.4% (2)	4% (1)	3% (7)
Shiv Sena	2.7% (7)	3.6% (7)	0% (0)	2.6% (6)	3.7% (1)	0% (0)	3% (7)
Biju Janata Dal	2.3 % (6)	2% (4)	3.3% (2)	2.2% (5)	3.7% (1)	8% (2)	1.7% (4)

Formatted Table

Samajwadi party	2.3% (6)	3% (6)	0% (0)	2.2% (5)	3.7% (1)	0% (0)	2.6% (6)
Dravida Munnetra Kazhagam	1.9% (5)	1% (2)	5% (3)	2.2% (5)	0% (0)	0% (0)	2.2% (5)
Others (24 political parties combined with each having less than 5 parliamentarians)	16.3% (42)	15.9% (31)	18.3% (11)	17.30% (40)	7.5% (2)	20% (5)	16% (37)
**Total**	**100%** **(257)**	**76.7%~~197~~** **(197)~~(76.7%)~~**	**23.3%** **(60)** **~~(23.3%)~~**	**89.5%~~230~~** **(23)** **~~(89.5%)~~**	**10.5%** **(27)** **~~(10.5%)~~**	**9.7%** **(25)** **~~(9.7%)~~**	**90.3%** **(232)** **~~(90.3%)~~**

Fig 2 Parliamentarians (state-wise) asking questions linking tobacco and ~~indigenous~~ Indigenous communities (1952-2022)

Fig 3 Timeline of the questions in relation to the major tobacco-related policy reforms (1952-2022)

Parliamentarians' concerns

We now describe parliamentarians' concerns about tobacco in relation to ~~indigenous~~ Indigenous communities in India using narratives and example questions under specific themes that emerged from analysis of the transcripts. Table 2 summarises these concerns by mentioning specific themes and issues within those themes.

Formatted: Left

Formatted: Left

Formatted: Left

Formatted: Left

Formatted: Left

Formatted: Space After: 10 pt

Table 2: Themes defining parliamentarians' concerns about tobacco and ~~indigenous~~ Indigenous communities in India

Themes defining parliamentarians' concerns	Specific aspects/issues within each theme
Health concerns	Specific health issues affecting bidi workers<br>● Tuberculosis<br>● Hunger<br>Access to healthcare services by workers and their families<br>● Hospitals for bidi workers<br>● Basic healthcare services for *tendu* leaf collectors<br>● Coverage (workers and their families) and implementation of the publicly funded health insurance scheme (RSBY)
Labour welfare concerns	Bidi worker's welfare fund<br>● Contributions by employers (bidi industry) towards bidi workers' welfare fund<br>● Effective allocation and utilisation of such funds by governments towards workers' welfare<br>Employees Provident Fund<br>● Facilitating enrolment of workers for the provident fund (social security)<br>● Irregularities by employers in depositing provident fund contributions<br>Identity cards for bidi workers<br>● Employers' role in recognising and issuing identity cards for workers<br>Contractualisation in the bidi industry<br>● Contract labour system in the bidi industry and exploitation of home-based bidi makers
Trade policies concerns	Collection and sale of *tendu* leaf<br>● ~~Indigenous~~ Indigenous communities' ownership rights over *tendu* leaf collection

	• Fair price to workers for sale of tendu leaf<br>Nationalization of tendu trade<br>• Potential impact on livelihoods and interests of tendu leaf pluckers<br>Foreign direct investment in cigarette sector<br>• Potential impact on bidi trade and hence workers within the bidi sector

Health concerns

The health of tobacco workers

While many parliamentarians asked questions related to tobacco-related health harms, these concerns were about the population in general and not specifically about ~~indigenous~~ Indigenous communities. The reference to ~~indigenous~~ Indigenous communities, in such debates, was incidental as part of government responses where specific policies/programs related to ~~indigenous~~ Indigenous communities and/or forest areas were mentioned.

Whenever health-related concerns were brought up by parliamentarians that specifically linked tobacco and ~~indigenous~~ Indigenous communities, they were typically framed around ~~indigenous~~ Indigenous community members as workers in tobacco supply chains, especially tendu-leaf collectors, bidi rollers and occasionally other aspects of tobacco supply chains (e.g., as tobacco barn owners or tobacco farmer/farm worker). Bidi is a smoked tobacco product, more prevalent than cigarettes in India, wherein processed tobacco is hand-rolled using tendu leaf (Diospyros melanoxylon) and tied with a cotton thread. ((248)

Some of the specific health concerns were related to inadequate nutrition and tuberculosis (with workers exposed to fine tobacco dust).

"…whether the Government is aware that the workers engaged in beedi making are suffering from the occupational hazard of T. B. Tuberculosis is permanently there among bidi workers. Lakhs of workers are affected by this but no medical care is provided to them…" (Oral Answers, Question 127, 1987)(2529)

The government cited experts' advice suggesting no factual basis to the widely perceived notion that tuberculosis is an occupational risk/hazard for bidi workers. (2529) Nonetheless, these concerns continued over time.

"…(a)whether there is high prevalence and mortality rate associated with tuberculosis among the women and bidi workers in the country…(d) the measures taken by the Government to control TB and funds spent therefor and the success achieved as a result thereof during the said period…" *(Unstarred Question 2886, Lok Sabha, 2012)(2630)*

The government responded by citing the Revised National Tuberculosis Control Program indicating that the program data do not point to higher rates of mortality from tuberculosis

Formatted: Justified

among (women) bidi workers and how the program universally promotes screening and access to free treatment for Tuberculosis to all including women and bidi workers.(26~~30~~)

There were concerns about hunger and undernutrition among bidi workers from ~~indigenous~~ Indigenous communities.

"...(a) whether Government's attention has been drawn to a new item captioned "Hunger stalks tribal beedi workers appearing in the Times of India" April 13, 1993? (b) if so, whether Government have obtained a report on the conditions of tribal beedi workers in Andhra Pradesh (c) what steps are proposed to prevent starvation amongst the section of labour? (Starred Question 11, Rajya Sabha, 1993)(27~~31~~)

The government responded by acknowledging that it has asked a report on this scenario from the state government of Andhra Pradesh.(27~~31~~)

Health services for workers and their families

Apart from specific health issues affecting workers, parliamentarians expressed concerns related to inadequate access to healthcare services or the functioning of publicly funded health insurance schemes for the workers and their families.

Given there ought to be specific policy providing physical healthcare delivery facilities for bidi workers, parliamentarians raised concerns about presence and functioning of such facilities over time.

"....(a) whether the Ministry has received a request for construction of hospital for Beedi workers at Gursahaiganj in Farrukhubad District; (b) if so, the decision taken thereon; (c) if no request has been received, whether Union Government propose suo moto to establish a hospital for these beedi workers in view of their long outstanding hardships on account of lack of basic medical facilities..." (Starred-Supplementary Question 127, Lok Sabha, 1987)(25~~9~~)

In this specific instance, the parliamentarian was also concerned about the delays in getting such a hospital in place despite repeated proposals. The government acknowledged the proposals for sanctioning a hospital and assured that it was under consideration while pointing out the expansion of dispensaries for bidi workers that was realised in the meantime.(25~~9~~)

Formatted: Justified

Such voices demanding healthcare services and/or accountability for functioning health insurance schemes for the bidi and tendu workers persisted over time.

"...(a) the number of tribal men and women in Orissa and Madhya Pradesh engaged to collect sal seeds, tendu leaves, Mahua and other forest and forests products...(c) whether any special programme for the health care of these tribal workers and their families have been formulated?" (Unstarred Question 4940, Rajya Sabha, 2000)(28~~32~~)

The government acknowledged that certain states (e.g., Andhra Pradesh and Madhya Pradesh) have come up with insurance for tendu leaf collectors and that Gujarat introduced a scheme to

provide compensation to tendu leaf collectors for any injuries or deaths during working seasons.(28~~32~~)

"...whether the Government is aware that RSBY centres in many districts are non-functional, if so, details thereof, State/ UT-wise, including Jharkhand along with the reasons therefor; and (e) whether the Government has taken note of irregularities in the above scheme being implemented in the interest of weavers in the country, if so, the details thereof and the action taken thereon along with the steps taken by the Government to increase its coverage particularly in tribal areas?" (Unstarred Question 4581, Lok Sabha, 2015)(29~~33~~)

The RSBY (*Rashtriya Swasthya Bima Yojana*, now integrated into the Pradhan Mantri Jan Arogya Yojana) referred to a national health insurance scheme initiated by the labour ministry (Government of India) that covered bidi workers as one of the 11 categories of workers apart from covering those living below the poverty line. The government clarified that there are no central government-run RSBY Centres as the scheme was being implemented by respective state governments.(29~~33~~) The government further clarified that no specific irregularities were found regarding implementing RSBY for handloom workers.(29~~33~~)

Labour welfare concerns

Many parliamentarians raised concerns about the realisation of labour rights and welfare measures for bidi workers asking for the accountability of government and employers in this regard.

Bidi workers' welfare fund

In 1976, the government of India enacted legislation that enabled the levy and collection of the cess on manufactured bidis towards creating a bidi workers' welfare fund(30~~4~~) as well as using such fund towards the welfare of bidi workers(31~~5~~) including the creation and improvements of public health and medical facilities, educational facilities for children of bidi workers, housing and recreational facilities, water supply and washing facilities and such other welfare measures.

Formatted: Justified

Parliamentarians were concerned about the bidi manufacturers paying adequate duty (cess) towards the bidi worker welfare fund.

"...(a) the amount collected as cess as on March 1995 from Bidi factories situated in tribal districts of Gujarat State; and (b) the amount incurred on the welfare for Beedi Workers during the above period?" (Unstarred question 6334. Lok Sabha, 1995)(32~~6~~)

"...(e) whether the Government are aware of irregularities being committed by major industrialists to escape from cess on Beedi; and (f) if so, the efforts being made by the Government in this regard?" (Unstarred Question 3101, Lok Sabha, 1995) (33~~7~~)

There were concerns about how effectively such funds were allocated and utilised by governments towards workers' welfare.

"...(a) whether the Government of Uttar Pradesh propose to allocate funds for the construction of houses of beedi workers particularly, in the tribal areas during the last three years." (Unstarred Question 1086, Lok Sabha 1998)(34~~8~~)

In these instances, the government assured to collect relevant information and disclose it to the upper house. The government assured that strict measures were being taken including the surprise checks of bidi companies by the Central Board of Excise and Customs to prevent cess evasion.

Provision of employee provident fund

Parliamentarians raised concerns about employers' role in enrolling and providing workers with the employee provident fund, a social security scheme allowing savings for the future wherein workers ought to get benefits on retirement, resignation, or death with a provision to also avail partial loans by workers for essential expenses. There were concerns that not all bidi workers were covered under the employees' provident fund.

"...(a) the number of the Beedi workers covered under the Employees Provident Fund Scheme in Andhra Pradesh in the tribal districts, (b) whether the amount deducted from the salaries of the Beedi workers are being properly credited to the Provident Fund Accounts; (c) whether a large number of the Beedi workers have not been covered under the Provident Fund Scheme, and (d) if so, the steps taken by the Government to check the malpractice?.." (Unstarred question 6530, Lok Sabha 1995)(35~~9~~)

The government responded by providing the overall number (including but not specific to tribal districts) of bidi workers enrolled under the Employees Provident Fund (a total of 4,10,512 bidi workers as on 28 February 1995). The government assured that it was recovering the amount being deducted from workers' wages by employers and was crediting it to workers' provident fund accounts.(35~~9~~)

"...(a) whether the government are aware of the complaints regarding fraudulent deposits in the provident funds of the beedi workers of Bihar and Gujarat due to which large scale resentment is prevailing among them..." and further seeking information about any actions taken by the government in this regard. (Question 6332, Lok Sabha, 1995)(36~~40~~)

In this instance, the government responded that the relevant information is being collected and will be disclosed to the house.

Identity cards for bidi workers

One of the basic requirements for receiving such welfare benefits is to get recognised as a bidi worker by having an identity card issued by the employer to a bidi worker as statutorily

mandated under the bidi Workers Welfare Fund Act. There were concerns about many workers not getting the identity cards.

"…(a) whether the Government have conducted any survey regarding the number of workers engaged in the Beedi Industry in certain States particularly in the tribal and backward areas of Gujarat State till September 30, 1995; (b) the number of identity card holders and non-identity card holders among them as on September, 1995, separately; (c) the efforts being made by the Government to liberate them from the harassment/oppression of contracts system…" (Unstarred Question 3101, Lok Sabha, 1995)(33~~37~~)

In this instance, the government assured to collect and disclose the information to the house.

Contractualization in the bidi industry

The contractors and sub-contractors started playing a major role for bidi companies, especially from the 1970s onward as bidi-making became largely a home-based work from what used to be a factory-based work. There remain concerns related to the exploitation of workers rolling bidis at home by the contractors.

"…(c) the efforts being made by the Government to liberate them from the harassment/oppression of contracts system…" (Unstarred Question 3101, Lok Sabha, 1995)(33~~7~~)

The government acknowledged the concern and conceded that given the historical developments resulting in the unique structure of the sector, it is "not feasible to abolish contract labour system" in the bidi sector.(33~~7~~) However, it assured that state governments have been requested to prevent exploitation of workers by ensuring better implementation of legal measures including, the beedi and cigar workers (conditions of employment) act 1966, the minimum wages act 1948, and formation of the tripartite committees (involving government, industry, and worker representatives) to address disputes and labour welfare issues.(33~~7~~)

Trade policy concerns

There were many concerns expressed by parliamentarians on how the government policies related to domestic and international trade would shape the domestic bidi industry in general (that include workers from ~~indigenous~~ Indigenous communities). These policies ranged from domestic decisions like taxation and nationalisation of tendu trade to measures like foreign direct investments in the tobacco sector. Here, we highlight concerns that specifically linked trade-related policies with the livelihoods of workers from ~~indigenous~~ Indigenous communities engaged in bidi rolling and tendu leaf collection.

Collection and sale of *tendu* leaf

Tendu leaf, largely collected by ~~indigenous~~ Indigenous communities from the central Indian forests, is an important ingredient in the bidi industry. It is used to wrap bidi tobacco. With the tightening of forest conservation laws, parliamentarians raised concerns about access to these forests by ~~indigenous~~ Indigenous communities for collecting tendu leaves that formed an important forest-dependent livelihood option for them.

"…(a) which minor forest produces would be purchased by Tribal Cooperative Marketing Development Federation to remove exploitation of the tribals by middlemen during the current financial year" (Unstarred Question 572, Rajya Sabha, 1990)(37~~41~~)

"….(a) whether the Common Minimum Programme of the Government vowed to give ownership right to Minor Forest produce (MFP) especially Tendu leaves to tribals; (b) if so, whether many States have changed track to give ownership right of minor forest produce except Tendu leaves; (c) if so, the reasons therefor; (d) whether the Government has taken up the matter with the Ministry of Environment & Forests to convince States to grant ownership right to Tribals of Tendu leaves…" (Unstarred Question 3321, Lok Sabha 2004)(38~~42~~)

The government highlighted that while ~~indigenous~~ Indigenous communities residing in and around forests were already granted usufructuary rights (rights allowing persons to use a property not owned by them) for collecting minor forest produce for domestic consumption, there were no "clear-cut ownership rights" on minor forest produce. It suggested that while this matter belongs to legislative business/mandate of state governments, the central government will request states to legislate conferring such ownership rights to ~~indigenous~~ Indigenous communities for minor forest produce including tendu leaves.(38~~42~~)

Nationalisation of *tendu* leaf trade

In the 1960s and 1970s, many state governments nationalised tendu leaf trade to enhance state revenues, prevent the exploitation of tendu leaf puckers and ensure supply for small- and medium-sized industries. When a parliamentarian raised concern on whether nationalisation will hurt ~~indigenous~~ Indigenous community members engaged in tendu leaf lucking, the government reiterated the protective impact of nationalisation on workers' rights.

"…(a) whether Government are aware that the main profession of Adivasis in Uttar Pradesh in Mirzapur district is Tendu leaves; (b) whether Government are aware of numerous sufferings of these Adivasis due to nationalisation of tendu leave trade; and (c) whether Government propose to de-nationalise the tendu leave trade for the benefit of the weaker sections of society i.e. Adivasis and if so. the salient features of the proposal?" (Unstarred Question 1008, Lok Sabha, 1977) (~~(4~~39~~3~~)

The government clarified that tendu leaf collection was a seasonal activity (typically from April to June) only partially contributing to the livelihoods of ~~indigenous~~ Indigenous communities.

It further denied the parliamentarian's claim about its exploitative impact and reiterated that the very intent of nationalising tendu leaf trade has been to safeguard the interests of tendu leaf collectors as they were not getting proper wages from contractors before such a move.(39~~43~~) It clarified that the government was not considering any proposals to de-nationalise the trade.(39~~43~~)

Parliamentarians kept raising concerns about state governments not buying tendu leaves from ~~indigenous~~ Indigenous communities and/or not remunerating them adequately for such purchases.

"….(a) whether millions of tribals in several districts of Madhya Pradesh are struggling for survival because of State Government refusal to purchase of tendu leaves from the tribals; (b) if so, the reasons therefore; and (c) the beneficiary oriented schemes introduced for providing alternate sources of incomes to the tribals displaced and deprived of collecting tendu leaves, sandalwood, etc." (Unstarred Question 1024, Lok Sabha 1992)(~~4~~40)

In this instance, the government assured to collect information on this from respective state governments and disclose it to the house. It indicated that efforts are being made to promote local employment of ~~indigenous~~ Indigenous communities residing in and around forests as part of eco-development, nature conservation and afforestation initiatives/schemes.(40~~4~~) In response to other related questions by parliamentarians, the government indicated that tendu leaf was considered one of the important minor forest produce to be promoted by the Tribal Co-operative Marketing Development Federation of India. (37~~41~~)

Foreign direct investment in the cigarette sector

Parliamentarians also wondered if the promotion of foreign direct investment in the cigarette industry would negatively impact the domestic bidi industry and hence in turn the manual labour within the bidi industry (that includes members from ~~indigenous~~ Indigenous community).

"…(a) whether the Government have reviewed its move to allow 100% Foreign Direct Investment (FDI) in cigarette industry; (b) if so, the major advantages of the economy by permitting such investment; (c) the extent to which it is likely to go against the interests of ~~indigenous~~ Indigenous bidi industry especially cottage industry and the workers engaged therein;…" (Starred Question 148, Lok Sabha,1999)(41~~5~~)

The government clarified that there was no ceiling in terms of foreign direct investment in cigarettes earlier. The government felt that cigarettes and bidis as different products and have very different market segments and hence enhanced foreign direct investments in the cigarette sector will only increase competitiveness in the cigarette sector without affecting the bidi industry or bidi workers.(41~~5~~)

Despite government assurances, parliamentarians ~~kept~~ expressed~~ing~~ concerns about the potential exploitation of ~~indigenous~~ Indigenous community members in tobacco supply chains, demanding policy measures from the government to safeguard workers' interests. Such concerns were about tendu leaf collectors, bidi workers as well as a minority of people who owned tobacco barns (facilities to cure tobacco leaves).

"…(a) whether it is fact that ITDA in Andhra Pradesh has disbursed funds for tobacco barn construction in tribal areas of West and East Godavari districts in 1990, 1991 and 1992 (b) The details of funds distributed in the form of loans and in the form of subsidy to tribal tobacco farmers in the West Godavari district (c) Whether any monitoring has been done to ensure that the tobacco barns said to be owned by the tribals have not been taken over by the non-tribals and (d) in how many instances have been taken cases where such barns were constructed only with the view to draw subsidy?.." (Unstarred Question 4234, Rajya Sabha, 1994)(42~~6~~)

"Whether Tendu leaf pluckers, most of whom are tribals in Madhya Pradesh, Chhattisgarh and Orissa, have demanded their share from the commercial exploitation of Tendu leaf and bamboos;…" (Unstarred Question 1886, Lok Sabha, 2002) (43~~8~~)

"…(a) which minor forest produces would be purchased by Tribal Cooperative Marketing Development Federation to remove exploitation of the tribals by middlemen during the current financial year" (Unstarred Question 572, Rajya Sabha, 1990) (44~~9~~)

Discussion

In this paper, through analysis of parliamentary questions over time (1952-2022), we describe major concerns that parliamentarians have expressed about tobacco in relation to ~~indigenous~~ Indigenous communities. We find that, overall, such concerns linking tobacco and ~~indigenous~~ Indigenous communities constitute a very small part (284 questions in the 70-year period from 1952 to 2022) of the total tobacco-related questions by parliamentarians (1315 questions only in the 20-year period from 1999 to 2019)~~ ~~(15~~27~~). Furthermore, a ~~very~~ tiny proportion of parliamentarians expressing concerns linking tobacco and ~~indigenous~~ Indigenous communities came from ~~indigenous~~ Indigenous communities. Their concerns were largely about marginal workers from ~~indigenous~~ Indigenous communities in tobacco supply chains: about their health issues and access to healthcare services, their exploitation by employers and potential threats to their interests and already precarious livelihoods. We now discuss some of these concerns by parliamentarians in the context of what we know from empirical studies on these issues as well as their implications for tobacco control and public health measures among ~~indigenous~~ Indigenous communities.

Invisible and precarious workforce within tobacco supply chains

Parliamentarians' concerns bring the spotlight on members of ~~indigenous~~ Indigenous communities that form a precarious workforce within tobacco supply chains (especially as bidi makers, tendu leaf collectors and small-holder tobacco farmers), something that is hardly

discussed in tobacco control discourse in India. Many studies corroborate concerns raised by parliamentarians about occupational risks and health hazards associated with such work as well as economic exploitation and poor state of labour rights/welfare of these workers despite the presence of specific regulatory provisions protecting these workers.(45-47~~50–52~~) Studies highlight that members of ~~indigenous~~ Indigenous communities often take to these works in the absence of alternative livelihood opportunities including lack of, or poor implementation of, public schemes assuring employment guarantees.(48~~53~~,49~~54~~)

While the WHO Framework Convention on Tobacco Control (a treaty signed and fully ratified by the Government of India) recognises the importance of promoting economically viable and healthy alternatives to tobacco-dependent livelihoods, there has been very limited progress on this front.(50,51~~55,56~~) This study, along with corroborating evidence discussed earlier, implies the need for a just transition wherein the tobacco supply reduction measures shall go hand in hand with measures that ensure fair remuneration and work conditions for the marginal workers in tobacco supply chains and that promote access to economically viable and acceptable livelihoods alternatives.

Health concerns are largely limited to the 'worker identity'

What is intriguing is that parliamentarians' concerns about health issues including access to healthcare services are primarily about workers from ~~indigenous~~ Indigenous communities engaged in tobacco supply chains. We did not come across any concerns about tobacco use and its health harms among ~~indigenous~~ Indigenous communities in general, beyond as workers in tobacco sector. This is despite the fact that parliamentarians have been increasingly concerned about the health harms of tobacco in general and tobacco use has remained disproportionately high among many ~~indigenous~~ Indigenous communities, who also experience poorer access to healthcare services/support. This could possibly imply that parliamentarians are yet to recognise and prioritise the challenge of substance use (especially tobacco use in this case) in addressing the health of ~~indigenous~~ Indigenous communities. This could also reflect a poor representation of ~~indigenous~~ Indigenous communities in the Indian parliament. The fact that the ~~indigenous~~ Indigenous communities have experienced the least decline in tobacco use over the last few decades, it is important to develop a special focus on ~~indigenous~~ Indigenous communities within tobacco control efforts, making them specifically relevant, acceptable, and informed by voices from ~~indigenous~~ Indigenous communities. ~~In this respect, the recent efforts in this direction including the first-of-its-kind national conference on tobacco use among tribal communities by the All India Institute of Medical Sciences (Deoghar)(57) as well as our efforts at understanding historical and socio-cultural pathways of substance use among indigenous communities at the Centre for Training, Research and Innovations in Tribal Health(20) are relevant. However, there is a long way to go, and the agenda needs to be about developing acceptable and effective interventions beyond simply expanding the existing approaches to tribal areas.~~

Tobacco's impact on forests and environment: a missing link?

Like the health harms of tobacco consumption, the negative impact of tobacco on forests and the environment seems to be another, more indirect, link to the health and wellbeing of ~~indigenous~~ Indigenous communities. The forests remain central to the survival and sustenance of forest-dwelling ~~indigenous~~ Indigenous communities. Tobacco, throughout its lifecycle, is known to have a negative impact on the environment and forests.(52~~8~~) In India, we have only started to map the magnitude of this problem: be it deforestation from tendu harvesting-related forest fires(53~~9~~) and illegal logging of fuelwood for tobacco curing(54~~60~~) or the environmental pollution resulting from tobacco production, consumption, and hazardous tobacco product waste.(55~~61~~)

Our findings and their discussion above have some implications for actions and further research related to tobacco control among ~~Indigenous~~ Indigenous communities in India. Overall, it highlights the limited but diverse political concerns linking tobacco and ~~Indigenous~~ Indigenous communities in India. We find that among the Indian Parliamentarians, but also in tobacco control discourse in general, there is very limited recognition of health consequences from the high overall tobacco use among many ~~Indigenous~~ Indigenous communities, and the growing social disparity in tobacco use. It is desirable to develop a special focus on ~~indigenous~~ Indigenous communities within tobacco control efforts, making such efforts relevant, acceptable, and informed by voices from ~~indigenous~~ Indigenous communities. In this respect, we have seen a few examples in recent past including a webinar series and the first-of-its-kind national conference on tobacco use among tribal communities by the All India Institute of Medical Sciences (Deoghar)(56~~7~~) as well as the efforts at understanding historical and socio-cultural pathways of substance use and creating context-specific tobacco-cessation support among ~~indigenous~~ Indigenous communities through the Centre for Training, Research and Innovations in Tribal Health(14~~20~~). However, we need to do more including enhanced implementation, using context-specific strategies,~~.~~ of the national tobacco control program in districts with ~~Indigenous~~ Indigenous communities.

The other important implication is to check the prevailing labour exploitation of ~~Indigenous~~ Indigenous community workers, especially in bidi supply chains, while strengthening efforts in providing alternative (non-tobacco) livelihoods options that are safer and viable. While there have been efforts to promote alternative crops in place of tobacco, such efforts have specifically targeted growers of cigarette tobacco.(57) There do not seem to be major efforts from governments to promote alternatives for marginal workers in bidi industry. Prioritising them under the prevailing wage employment promotion initiatives like the Mahatma Gandhi Rural Employment Guarantee Scheme(58) and the Pradhan Mantri Kaushal Vikas Yojana (Skill India Mission)(59) can help in this direction. While our study was limited to p~~P~~arliamentary concerns, the future studies could look at other sources (such as news media, social movements, civil society voices) to map the broader public/policy discourse linking tobacco and ~~Indigenous~~ Indigenous communities in India. It is time that the public health research and advocacy community expand their remit to engage with complex and multiple ways in which tobacco ~~use~~use, and the tobacco industry are impacting ~~indigenous~~ Indigenous communities to make any ~~positive~~ equitable difference.

Formatted: Justified, Space After: 0 pt

Limitation

There are limitations, mainly arising out of the data, to our study. The digital search catalogues of the lower and the upper houses of the Indian parliament that we used to source the transcripts are well-optimised for specific searches as they do not recognise Boolean operators. There is no specific guidance provided to optimise their use. This could have limited the number of relevant transcripts we could source despite the elaborate searches we ran. Apart from the low representation (in terms of absolute numbers) of the indigenous Indigenous communities in the Indian parliament, the study of the parliamentary proceedings over 30 years (1980-2009) by Ayyangar and Jacob(6062) reveals that parliamentarians from scheduled tribe category, as well as women parliamentarians, participate less actively (asking a lesser number of questions on an average) in the Question Hour compared to men and parliamentarians from upper castes. This is an important limitation as the voices of parliamentarians from indigenous Indigenous communities remain limited and so are the voices of women (most bidi makers and many of the tendu leaf collectors are women). They also highlight that participation of parliamentarians from the northeasternnorth-eastern states of India remainsremain limited – these are the states with a greater proportion of indigenous Indigenous communities as well as a high prevalence of tobacco use.(62) While we included all the question types, we did not include the debates – a longform transaction specifically around legislative developments. While it is a limitation, it is also less likely that it would have added any substativesubstantive data given that tobacco-related legislations do not have specific emphasis on Indigenous Indigenous communities.

The parliamentary questions are a are important in terms of timeline dataset; however, it is limited component of the broader policy discourse. Furthermore. While our analysis focuses on solely on parliamentary questions, we understand there are many other dataset and sources that could offer similar ground for analysis. The parliamentary questions are that are reviewed are typically factual and concise but does not offer in-depth insigdevoid of candid detailshts. Another important aspect is that the parliamentary questionsThese questions are often framed with many considerations (like party agenda, political signalling, personal priority, individual interests) and do not always reflect genuine concerns of constituents. , etc. Thus, it may be coded with various factors that can be political or personal to the parliamentarians. This is rather difficult to discuss such factors and challenging not to acknowledge it. Another methodological limitation can be not including the parliamentary debates in this paper. The debates that happen occasionally might have provided deeper insight into the broader context of policy discussion and helped in analysing the complexity of legislative process surrounding tobacco and indigenous communities in India.

Finally, the very procedure for and the nature of proceedings during the Question Hour are such that puts a limit to the length and candid nature of transactions among parliamentarians.

Conclusions

Overall, the representation of indigenous Indigenous communities in the Indian parliament remains very limited. Parliamentarians, over time, expressed varied concerns linking tobacco and indigenous Indigenous communities in India. The major concerns included (1)

Formatted: Font: 12 pt

occupational and other health hazards as well as inadequate access to healthcare services faced by members from ~~indigenous~~ Indigenous communities working as bidi makers and tendu leaf collectors; (2) unfair wages, exploitative work conditions and inadequate social security to these workers; and (3) perceived negative impact on tobacco-linked livelihoods resulting from trade and investment related policies in the tobacco sector. Parliamentarians did not raise issues related to high tobacco use and tobacco-related harms among ~~indigenous~~ Indigenous communities as well as the negative impact of tobacco on forests that are central to the lives of ~~indigenous~~ Indigenous communities.

Acknowledgement

We would like to acknowledge Soniya SM, pursuing her public health degree at the Karnataka State Rural Development and Panchayat Raj University, for her support in data management during her three-month internship with us.

Funding statement

This work is supported by the DBT Wellcome Trust India Alliance CRC grant [Grant No. IA/CRC/20/1/600007] awarded to Prashanth NS, Suresh Shapeti, Deepa Bhat, and Upendra Bhojani. Upendra Bhojani was also supported through the DBT Wellcome Trust India Alliance (clinical and public health) Senior Fellowship awarded to him [Grant No. IA/CPH/22/1/506533].

References

1. Kaur J, Rinkoo AV, Richardson S. Update on numbers of tobacco-attributable deaths by country in the South-East Asia region: implications for policy. Tob Control. 2024;0:1–4. [cited 2025 July 10] doi:10.1136/tc-2024-058599
2. Ministry of Health and Family Welfare & Ministry of Tribal Affairs. Government of India. Report of the expert committee on tribal health. Tribal health in India: bridging the gap and a road map for the future. 2018. [cited 2025 July 10] Available from: https://tribalhealthreport.in/full-report/
3. Murmu J, Agrawal R, Manna S, et al. Social determinants of tobacco use among tribal communities in India: evidence from the first wave of Longitudinal Ageing Study in India. PLOS One 2023 [cited 2025 September 28] Available from: https://doi.org/10.1371/journal.pone.0282487
4. Minichiello A, Lefkowitz ARF, Firestone M, Smylie JK, Schwartz R. Effective strategies to reduce commercial tobacco use in Indigenous communities globally: A systematic review. BMC Public Health 2015;16:21 [cited 2025 July 10] Available from: https://doi.org/10.1186/s12889-015-2645-x
5. Crocetti AC, Cubillo (Larrakia) B, Lock (Ngiyampaa) M, et al. The commercial determinants of Indigenous health and well-being: a systematic scoping review. BMJ Global Health 2022;7:e010366. [cited 2025 July 10] doi:10.1136/bmjgh-2022-010366
6. Maddox R, Waa A, Lee K, et al. Commercial tobacco and Indigenous peoples: a stock take on Framework Convention on Tobacco Control progress Tob

Formatted: Font: 18 pt, English (United Kingdom)
Formatted: Font: 12 pt, English (United Kingdom)
Formatted: Justified, Right: 0.2", Space After: 0 pt, Line spacing: single
Formatted: Space After: 0 pt, Line spacing: single
Formatted: Font: 12 pt
Formatted: Font: 12 pt
Formatted: Font: 12 pt
Formatted: Font: 12 pt
Formatted: Font: 12 pt
Formatted: Font: 12 pt
Formatted: Font: 12 pt
Formatted: Font: 12 pt
Formatted: Font: 12 pt
Formatted: Font: 12 pt
Formatted: Font: 12 pt
Formatted: Font: 12 pt
Formatted: Font: 12 pt
Formatted: Font: 12 pt
Formatted: Font: 12 pt
Formatted: Font: 12 pt
Formatted: Font: 12 pt
Formatted: Font: 12 pt

Control 2019;28:574-581. [cited 2025 July 10] Available from: https://tobaccocontrol.bmj.com/content/28/5/574

7. Commonwealth of Australia. National Drug Strategy 2017-2026. Department of Health; 2017 [cited 2025 July 10] Available from: https://www.health.gov.au/sites/default/files/national-drug-strategy-2017-2026.pdf

8. Government of Canada. Canada’s Tobacco Strategy. [cited 2025 July 10] Available from: https://www.canada.ca/en/health-canada/services/publications/healthy-living/canada-tobacco-strategy.html#a3

9. Government of India. Statistical profile of scheduled tribes in India 2013. Statistical Division, Ministry of Tribal Affairs, Government of India; 2013. [cited 2025 July 10] Available from: https://ruralindiaonline.org/en/library/resource/statistical-profile-of-scheduled-tribes-in-india-2013/

10. National Commission for Scheduled Tribes. Constitutional provisions for development of scheduled tribes. [cited 2025 July 10] Available from: https://socialwelfare.vikaspedia.in/viewcontent/social-welfare/scheduled-tribes-welfare/constitutional-provisions-for-development-of-scheduled-tribes?lgn=en

11. Ministry of Health & Family Welfare Government of India. Tobacco control laws and related laws in India. National Tobacco Control Program. 2024 [cited 2025 April 10]. Available from: https://ntcp.mohfw.gov.in/acts_rules_regulations

12. Ministry of Health & Family Welfare Government of India. GATS 2: Global adult tobacco survey, factsheet India 2016-17. [cited 2025 April 25] Available from: https://www.mohfw.gov.in/sites/default/files/GATS-2 FactSheet.pdf

13. Hebbar PB, Bhojani U, Schayck O Van, Babu G, Dsouza V. Shifting the gaze on implementation : examining the association between the implementation of tobacco control laws and prevalence of tobacco using data from a nationally representative survey. BMC Public Health. 2023;1–9. [cited 2025 July 10] Available from: https://doi.org/10.1186/s12889-023-16780-8

14. Institute of Public Health Bengaluru. Centre for Training, Research, and Innovation in Tribal Health (CTRITH). 2025. [cited 2025 July 10] Available from: https://iphindia.org/centre-for-training-research-and-innovation-in-tribal-health-ctrith/

15. Varma A, Chilgod L, Bhojani U. Diverse and competing interests around tobacco: Qualitative analysis of two decades of parliamentary questions in India. BMJ Glob Heal. 2021;6(4):1–10. [cited 2025 July 10] Available from: https://gh.bmj.com/content/6/4/e004093

16. Government of India. Ministry of Tribal Affairs. [cited 2025 July 10] Available from: https://tribal.nic.in/

17. General Information. Lok Sabha (House of the People). 2025. [cited 2025 July 10] Available from: https://sansad.in/ls/questions/questions

18. Introduction: Question Hour. Rajya Sabha (Council of States). 2025. [cited 2025 July 10] Available from: https://sansad.in/rs/questions/general-information

19. Loksabha. Questions and Answers. [cited 2025 July 10] Available from: https://sansad.in/ls/questions/questions-and-answers

20. Rajyasabha. Questions and Answers. [cited 2025 July 10] Available from: https://sansad.in/rs/questions/questions-and-answers

21. Anderson R. Thematic Content Analysis (QCA): descriptive presentation of qualitative data; 2007 [cited 2025 July 10] Available from: https://rosemarieanderson.com/wp-content/uploads/2014/08/ThematicContentAnalysis.pdf

22. Das SS. Traditional healing among the Tai Ahoms of Assam: changing dynamics and its interaction with the public health system. PhD Thesis. Jawaharlal Nehru University,

New Delhi; 2024.

23. Das SS. Witch-Hunting from a public health perspective: narratives from Assam. MPhil Thesis. Jawaharlal Nehru University, New Delhi; 2018.

24. Gupta PC, Asma S. Bidi smoking and public health. Ministry of Health Family Welfare. New Delhi; 2008. [cited 2025 July 10] Available from: https://ntcp.mohfw.gov.in/assets/document/Monograph on Bidi Smoking and Public Health.pdf

25. Parliament of India. Oral answers, Question 127. 1987. [cited 2025 July 10] Available from: https://eparlib.nic.in/bitstream/123456789/878354/1/08_IX_16-11-1987_p13_p15_s127.pdf

26. Parliament of India. Unstarred Question No: 2886. 2012. [cited 2025 July 10] Available from: https://eparlib.nic.in/bitstream/123456789/616866/1/118419.pdf

27. Parliament of India (Rajya Sabha). Starred Question 11. 1993. p. 39–40.

28. Parliament of India (Rajya Sabha). Question 4940. 2000. p. 87

29. Parliament of India (Lok Sabha). Unstarred Question 4581. 2016. [cited 2025 July 10] Available from: https://eparlib.nic.in/bitstream/123456789/688010/1/40331.pdf

30. the Beedi Workers Welfare Fund Act, 1976; 1976 p. 1–7.

31. Government of India. Beedi and Cigar Workers (Conditions of Employment) Act 1966. [cited 2025 July 10] Available from: https://www.indiacode.nic.in/handle/123456789/1675?sam_handle=123456789/1362

32. Parliament of India (Lok Sabha). Question 6334. 1995. p. 43. [cited 2025 July 10] Available from: https://eparlib.nic.in/bitstream/123456789/2392213/1/10_XIII_19-05-1995_p26_p26_u6332.pdf

33. Parliament of India (Lok Sabha). Question 3101. 1995. p. 485–6. [cited 2025 July 10] Available from: https://eparlib.nic.in/bitstream/123456789/2402879/1/10_XV_18-12-1995_p247_p247_u3101.pdf

34. Parliament of India (Lok Sabha). Question 1086. 1998. p. 305. [cited 2025 July 10] Available from: https://eparlib.nic.in/bitstream/123456789/2963091/1/US_1086_02-06-1998_1.pdf

35. Parliament of India (Lok Sabha). 1995. p. 174 Question 6530. [cited 2025 July 10] Available from: https://eparlib.nic.in/bitstream/123456789/2392411/1/10_XIII_19-05-1995_p91_p91_u6530.pdf

36. Parliament of India (Lok Sabha). Question 6332. 1995. p. 43. [cited 2025 July 10] Available from: https://eparlib.nic.in/bitstream/123456789/2392213/1/10_XIII_19-05-1995_p26_p26_u6332.pdf

37. Parliament of India (Rajya Sabha). Question 572. 1990.

38. Parliament of India (Lok Sabha). Question 3321. [cited 2025 July 10] 2004. Available from: https://eparlib.nic.in/bitstream/123456789/572298/1/7431.pdf

39. Parliament of India (Lok Sabha). Question 1008. 1977. p. 91.

40. Parliament of India (Lok Sabha). Question 1024. [cited 2025 July 10] 1992. p. 559. Available from: https://eparlib.nic.in/bitstream/123456789/2349633/1/10_IV_14-07-1992_p287_p287_u1024.pdf

41. Parliament of India (Lok Sabha). Question 148. Vol. 1920. 1999. p. 34–5. [cited 2025 July 10] Available from: https://eparlib.nic.in/bitstream/123456789/793655/1/12_IV_05031999_p21_p22_s148.pdf

42. Parliament of India (Rajya Sabha). Question 4324. 1994.

43. Parliament of India (Lok Sabha). Question 1886. 2002. [cited 2025 July 10] Available from: https://eparlib.nic.in/bitstream/123456789/433857/1/48211.pdf

44. Parliament of India (Rajya Sabha). Question 572. 1990.

45. Government of India. Evaluation study on the implementation of Minimum Wages Act, 1948 in beedi making industry in Madhya Pradesh. Chandigarh; 2015. [cited 2025 April 10] Available from: https://labourbureau.gov.in/uploads/pdf/Report_on_Beddi_Industry_2015.pdf

46. Status of tendu leaf pluckers in Orissa: a study of their socio-economic conditions with special reference to children and the system of bonded labour. Delhi; 2006. [cited 2025 April 10] Available from: http://hdsindia.org/wp-content/uploads/2018/06/NHRC-Tendu-Leaf-Pluckers-Report.pdf

47. Rathore A, P. Ugargol A. Beedi rolling and the health hazards associated with occupational exposure: a situational review from Karnataka, India. RGUHS Natl J Public Heal. 2018;4(1). [cited 2025 July 10] Available from: https://rnjph.journalgrid.com/view/article/rnjph/653

48. Sabar B, Nayak N, Achoth L. Tendu leaves collection in India: livelihoods, rights and challenges for alternative to tobacco - evidences from five Indian states. J Gov Public Policy. 2016;6(1):106-123.

49. Russell A, Chandra P, Robson M, Narayanan P, Joseph S, Mukherjee P, et al. Implementing FCTC Article 17 Through Participatory Research With Bidi Workers in Tamil Nadu, India. Nicotine Tob Res. 2022;24(11):1714–9. [cited 2025 July 10] Availabe from: https://doi.org/10.1093/ntr/ntac075

50. World Health Organization. WHO Framework Convention on Tobacco Control. Geneva; 2003. [cited 2025 July 10] Available from: https://fctc.who.int/resources/publications/i/item/9241591013

51. Chung-Hall J, Craig L, Gravely S, Sansone N, Fong GT. Impact of the WHO FCTC over the first decade: A global evidence review prepared for the Impact Assessment Expert Group. Tob Control. 2019;28:S119–28. [cited 2025 July 10] Available from: https://tobaccocontrol.bmj.com/content/28/Suppl_2/s119

52. World Health Organization. Tobacco and its environmental impact: an overview. 2017 [cited 2025 April 10] Available from: http://dx.doi.org/10.1016/j.ccm.2013.09.011

53. Singh M, Sah P, Kumar P. Forest fires and tendu leaf collection in India: an evidence-based analysis. New Delhi; 2023. [cited 2025 April 10] Available from: https://iforest.global/research/report_forest-fires-and-tendu-leaf-collection-in-india-an-evidence-based-analysis-request-download/

54. Nayak NS. Tobacco Curing and Fuel Efficiency in Karnataka, India. Kathmandu; 2013. [cited 2025 July 10] Available from: http://www.sandeeonline.org/uploads/documents/publication/1007_PUB_Working_Paper_77_Nayantara.pdf

55. All India Institute of Medical Sciences Jodhpur, Indian Council of Medical Research - National Institute of Cancer Prevention and Research, The International Union against Tuberculosis and Lung Diseases. The Environmental Burden of Tobacco Products Wastes in India: tobacco use makes you ugly, your habitate uglier. 2022. [cited 2025 April 10] Available from: https://nicpr.org/wp-content/uploads/2023/02/tobacco_waste_report.pdf

56. All India Institute of Medical Sciences (Deoghar). 1st National Conference on "Tobacco Use among Tribal Communities" 20-21 July 2024. [cited 2025 April 10] Deoghar; 2024. Available from: https://www.aiimsdeoghar.edu.in/Content/resources/document/notice/national_conference_tutc_2024_19062024_050827183.pdf

57. Government of India. Reduction in tobacco production in India. Press Information Bureau. Ministry of Commerce & Industry; 2019 [cited 2025 July 10] Available from: https://www.pib.gov.in/newsite/PrintRelease.aspx?relid=191176

58. Government of India. Mahatma Gandhi National Rural Employment Guarantee Scheme. Ministry of Rural Development, Government of India; 2025. [cited 2025 July 10] Available from: https://nrega.dord.gov.in/MGNREGA_new/Nrega_home.aspx
59. Government of India. Pradhan Mantri Jaushal Vikas Yojana. Ministry of Skill Development and Entrepreneurship, Government of India. [cited 2025 July 10] Available from: https://www.msde.gov.in/offerings/schemes-and-services/details/pradhan-mantri-kaushal-vikas-yojana-4-0-pmkvy-4-0-2021-ITO3ATMtQWa
60. Ayyangar S, Jacob S. Studying the indian legislature: What does question hour reveal? Stud Indian Polit. 2014;2(1):1–19. [cited 2025 July 10] doi: 10.1177/2321023014526023

1. Kaur J RARS. Update on numbers of tobacco-attributable deaths by country in the South-East Asia region: implications for policy. Tob Control 2025 Jul 31;34(4):544-547 doi: 101136/tc-2024-058599 . 2025 Jul 31;34(4):544–7.
2. Tribal Health: Bridging the gap and road map for the future. Report of the Expert Committee on Tribal Health. New Delhi: Government of India; 2018. Available from: National Health Systems Resource Centre website. [Internet]. 2018 [cited 2025 Sep 9]. Available from: https://nhm.gov.in/nhm_components/tribal_report/Executive_Summary.pdf
3. DBT/Wellcome Trust India Alliance. 11th India Alliance annual conclave 2023: abstract book. Hyderabad: DBT/Wellcome Trust India Alliance; 2023. 165 p. . 2023.
4. Glover M. Smoking Prevalence Among Indigenous Peoples of the World. 2021; Available from: https://www.researchgate.net/publication/350538235
5. Martell B, GB, & CR. Disparities in Adult Cigarette Smoking — United States, 2002–2005 and 2010–2013. . MMWR Morb Mortal Wkly Rep 65, . 2016;753–8.
6. Colonna E, Maddox R, Cohen R, Marmor A, Doery K, Thurber K A, et al. Review of tobacco use among Aboriginal and Torres Strait Islander peoples. Australian Indigenous HealthBulletin ISSN 1445-7253 From the Australian Indigenous HealthInfoNet [Internet]. 2020 Apr;20(No 2). Available from: https://aodknowledgecentre.ecu.edu.
7. Jetty R. Tobacco use and misuse among Indigenous children and youth in Canada. Paediatrics and Child Health (Canada). 2017 Oct 1;22(7):395–9.
8. Minichiello A, Lefkowitz ARF, Firestone M, Smylie JK, Schwartz R. Effective strategies to reduce commercial tobacco use in Indigenous communities globally: A systematic review Health behavior, health promotion and society. Vol. 16, BMC Public Health. BioMed Central Ltd.; 2016.

Formatted: Font: 12 pt

Formatted: Font: 12 pt

Formatted: Font: 12 pt

Formatted: Space After: 0 pt, Line spacing: single

Formatted: Font: 12 pt

Formatted: Space After: 0 pt, Line spacing: single

9. Maddox R, Telford RM, Waa A, Diaz A, Bradbrook SK, Calma T, et al. Eradication of commercial tobacco related disease and death. Tob Control. 2024;
10. INDIGENOUS PEOPLES' COUNTRY PROFILE INDIA [Internet]. Available from: https://tribal.nic.in/ST/Tribal%20Profile.pdf,
11. Pande R. Globalisation and Women's Work in the Beedi Industry. Stud Hist Oecon. 2019 Dec 1;37(1):191–221.
12. Tyagi J, Beri D, Ingale S, Sinha P, Bhaumik S. Occupational health hazards of bidi workers and their families in India: a scoping review. BMJ Glob Health. 2023 Nov 2;8(11).
13. Shaikh S, Krishna N, Ghosh K, Chawale SV, Pal J, Harit L. Occupation-related morbidity patterns and their determinants among beedi workers in Murshidabad District, West Bengal. Int J Community Med Public Health. 2025 Jun 30;12(7):3202–10.
14. Varma A, Chilgod L, Bhojani U. Diverse and competing interests around tobacco: Qualitative analysis of two decades of parliamentary questions in India. BMJ Glob Health. 2021 Apr 7;6(4).
15. Glover M. Lack of Smoking Prevalence Data for Indigenous Peoples Worldwide Contributes to Epidemiological Invisibility. Data Sci J [Internet]. 2025;1. Available from: https://doi.org/10.5334/dsj-
16. Nath S, Narasimha VL, Biswas B, Kumar U, Jahnavi G, Ayub A, et al. Tobacco Use Among Tribal Communities in India: A Systematic Review and Meta-Analysis of Prevalence Studies. Cureus. 2025 Jul 14;
17. Ministry of Health & Family Welfare Government of India. [Internet]. 2024 [cited 2025 Sep 9]. Tobacco control laws and related laws in India. National Tobacco Control Program. Available from: [cited 2025 April 10]. Available from: https://ntcp.mohfw.gov.in/acts_rules_regulations
18. Ministry of Health & Family Welfare Government of India. GATS 2: Global adult tobacco survey, factsheet India 2016-17. 2017.
19. Hebbar PB, Bhojani U, Schayck O Van, Babu G, Dsouza V. Shifting the gaze on implementation : examining the association between the implementation of tobacco control laws and prevalence of tobacco using data from a nationally representative survey. BMC Public Health. 2023;1–9.
20. Institute of Public Health Bengaluru. Centre for Training, Research, and Innovation in Tribal Health (CTRITH) [Internet]. 2025. Available from: https://iphindia.org/centre-for-training-research-and-innovation-in-tribal-health-ctrith/
21. Lok Sabha (House of the People) [Internet]. 2025. General Information. Available from: https://sansad.in/ls/questions/questions
22. Rajya Sabha (Council of States) [Internet]. 2025. Introduction: Question Hour. Available from: https://sansad.in/rs/questions/general-information
23. Attride-Stirling J. Thematic networks: an analytic tool for qualitative research. Qualitative Research . 2021;1(3):385–405.
24. Dsouza R, Bhojani U. Strategic and contested use of food laws to ban smokeless tobacco products in India: a qualitative analysis of litigation. Tob Control. 2023 May 1;32(3):275–9.
25. Das SS. Traditional healing among the Tai Ahoms of Assam: changing dynamics and its interaction with the public health system. PhD Thesis. Jawaharlal Nehru University, New Delhi; 2024.
26. Witch-Hunting from a public health perspective: narratives from Assam. MPhil Thesis. Jawaharlal Nehru University, New Delhi; 2018.

27. Varma A, Chilgod L, Bhojani U. Diverse and competing interests around tobacco: Qualitative analysis of two decades of parliamentary questions in India. BMJ Glob Health. 2021;6(4):1–10.
28. Gupta PC, Asma S. Bidi smoking and public health. Ministry of Health Family Welfare. New Delhi; 2008.
29. Parliament of India. Oral answers, Question 127 [Internet]. 1987. p. 20. Available from: chrome-extension://efaidnbmnnnibpcajpcglclefindmkaj/https://eparlib.nic.in/bitstream/123456789/878354/1/08_IX_16-11-1987_p13_p15_s127.pdf
30. Government of India. Unstarred Question No: 2886 [Internet]. 2012. Available from: https://eparlib.nic.in/bitstream/123456789/616866/1/118419.pdf
31. Parliament of India (Rajya Sabha). Starred Question 11. 1993. p. 39–40.
32. Parliament of India (Rajya Sabha). Question 4940. 2000. p. 87.
33. Parliament of India (Lok Sabha). Unstarred Question 4581 [Internet]. 2016. Available from: https://eparlib.nic.in/bitstream/123456789/688010/1/40331.pdf
34. the Beedi Workers Welfare Fund Act , 1976. 1976 p. 1–7.
35. Government of India [Internet]. 1966. p. 1–15 the Beedi and Cigar Workers (Conditions of Employment) Act , 1966. Available from: https://www.indiacode.nic.in/handle/123456789/1675?sam_handle=123456789/1362
36. Parliament of India (Lok Sabha). Question 6334 [Internet]. 1995. p. 43. Available from: https://eparlib.nic.in/bitstream/123456789/2392213/1/10_XIII_19-05-1995_p26_p26_u6332.pdf
37. Parliament of India (Lok Sabha). Question 3101 [Internet]. 1995. p. 485–6. Available from: https://eparlib.nic.in/bitstream/123456789/2402879/1/10_XV_18-12-1995_p247_p247_u3101.pdf
38. Parliament of India (Lok Sabha). Question 1086 [Internet]. 1998. p. 305. Available from: https://eparlib.nic.in/bitstream/123456789/2963091/1/US_1086_02-06-1998_1.pdf
39. Parliament of India (Lok Sabha). 1995. p. 174 Question 6530. Available from: https://eparlib.nic.in/bitstream/123456789/2392411/1/10_XIII_19-05-1995_p91_p91_u6530.pdf
40. Parliament of India (Lok Sabha). Question 6332 [Internet]. 1995. p. 43. Available from: https://eparlib.nic.in/bitstream/123456789/2392213/1/10_XIII_19-05-1995_p26_p26_u6332.pdf
41. Parliament of India (Rajya Sabha). Question 572. 1990.
42. Parliament of India (Lok Sabha). Question 3321 [Internet]. 2004. Available from: https://eparlib.nic.in/bitstream/123456789/572298/1/7431.pdf
43. Parliament of India (Lok Sabha). Question 1008. 1977. p. 91.
44. Parliament of India (Lok Sabha). Question 1024 [Internet]. 1992. p. 559. Available from: https://eparlib.nic.in/bitstream/123456789/2349633/1/10_IV_14-07-1992_p287_p287_u1024.pdf
45. Parliament of India (Lok Sabha). Question 148 [Internet]. Vol. 1920. 1999. p. 34–5. Available from: https://eparlib.nic.in/bitstream/123456789/793655/1/12_IV_05031999_p21_p22_s148.pdf
46. Parliament of India (Rajya Sabha). Question 4324. 1994.

47. Parliament of India (Lok Sabha). Question 1886 [Internet]. 2002. Available from: https://eparlib.nic.in/bitstream/123456789/433857/1/48211.pdf
48. Parliament of India (Lok Sabha). Question 1886. 2002. [cited 2025 April 10] Available from: https://eparlib.nic.in/bitstream/123456789/433857/1/48211.pdf.
49. Parliament of India (Rajya Sabha). Question 572. 1990.
50. Government of India. Evaluation study on the implementation of Minimum Wages Act , 1948 in beedi making industry in Madhya Pradesh. Chandigarh; 2015.
51. Status of tendu leaf pluckers in Orissa: a study of their socio-economic conditions with special reference to children and the system of bonded labour. Delhi; 2006.
52. Rathore A, P. Ugargol A. Beedi rolling and the health hazards associated with occupational exposure: A situational review from Karnataka, India. RGUHS National Journal of Public Health. 2018;4(1).
53. Sabar B, Nayak N, Achoth L. Tendu Leaves Collection in India: Livelihoods, Rights and Challenges for Alternative to Tobacco- Evidences from Five Indian States. Journal of Governance and Public Policy. 2016;6(1):5–6.
54. Russell A, Chandra P, Robson M, Narayanan P, Joseph S, Mukherjee P, et al. Implementing FCTC Article 17 Through Participatory Research With Bidi Workers in Tamil Nadu, India. Nicotine and Tobacco Research. 2022;24(11):1714–9.
55. World Health Organization. WHO Framework Convention on Tobacco Control. Geneva; 2003.
56. Chung-Hall J, Craig L, Gravely S, Sansone N, Fong GT. Impact of the WHO FCTC over the first decade: A global evidence review prepared for the Impact Assessment Expert Group. Tob Control. 2019;28:S119–28.
57. All India Institute of Medical Sciences (Deoghar). 1st National Conference on "Tobacco Use among Tribal Communities" 20-21 July 2024. Vol. 2024. Deoghar; 2024.
58. World Health Organization. Tobacco and its environmental impact: an overview. Vol. 34. 2017.
59. Singh M, Sah P, Kumar P. Forest fires and tendu leaf collection in India: an evidence-based analysis. New Delhi; 2023.
60. Nayak NS. Tobacco Curing and Fuel Efficiency in Karnataka, India. Kathmandu; 2013.
61. SPH AIIMS Jodhpur, ICMR_NICPR, The Union. The Environmental Burden of Tobacco Products Wastes in India: tobacco use makes you ugly, your habitate uglier. 2022.
62. Ayyangar S, Jacob S. Studying the indian legislature: What does question hour reveal? Studies in Indian Politics. 2014;2(1):1–19.

References

1. Kaur J RARS. Update on numbers of tobacco-attributable deaths by country in the South-East Asia region: implications for policy. Tob Control 2025 Jul 31;34(4):544-547 doi: 101136/tc-2024-058599 . 2025 Jul 31;34(4):544–7.
2. Tribal Health: Bridging the gap and road map for the future. Report of the Expert Committee on Tribal Health. New Delhi: Government of India; 2018. Available from: National Health Systems Resource Centre website. [Internet]. 2018 [cited 2025 Sep 9]. Available from: https://nhm.gov.in/nhm_components/tribal_report/Executive_Summary.pdf

3. DBT/Wellcome Trust India Alliance. 11th India Alliance annual conclave 2023: abstract book. Hyderabad: DBT/Wellcome Trust India Alliance; 2023. 165 p. . 2023.
4. Glover M. Smoking Prevalence Among Indigenous Peoples of the World. 2021; Available from: https://www.researchgate.net/publication/350538235
5. Martell B, GB, & CR. Disparities in Adult Cigarette Smoking — United States, 2002–2005 and 2010–2013. . MMWR Morb Mortal Wkly Rep 65, . 2016;753–8.
6. Colonna E, Maddox R, Cohen R, Marmor A, Doery K, Thurber K A, et al. Review of tobacco use among Aboriginal and Torres Strait Islander peoples. Australian Indigenous HealthBulletin ISSN 1445-7253 From the Australian Indigenous HealthInfoNet [Internet]. 2020 Apr;20(No-2). Available from: https://aodknowledgecentre.ecu.edu.
7. Jetty R. Tobacco use and misuse among Indigenous children and youth in Canada. Paediatrics and Child Health (Canada). 2017 Oct 1;22(7):395–9.
8. Minichiello A, Lefkowitz ARF, Firestone M, Smylie JK, Schwartz R. Effective strategies to reduce commercial tobacco use in Indigenous communities globally: A systematic review Health behavior, health promotion and society. Vol. 16, BMC Public Health. BioMed Central Ltd.; 2016.
9. Maddox R, Telford RM, Waa A, Diaz A, Bradbrook SK, Calma T, et al. Eradication of commercial tobacco related disease and death. Tob Control. 2024;
10. INDIGENOUS PEOPLES' COUNTRY PROFILE INDIA [Internet]. Available from: https://tribal.nic.in/ST/Tribal%20Profile.pdf,
11. Pande R. Globalisation and Women's Work in the Beedi Industry. Stud Hist Oecon. 2019 Dec 1;37(1):191–221.
12. Tyagi J, Beri D, Ingale S, Sinha P, Bhaumik S. Occupational health hazards of bidi workers and their families in India: a scoping review. BMJ Glob Health. 2023 Nov 2;8(11).
13. Shaikh S, Krishna N, Ghosh K, Chawale SV, Pal J, Harit L. Occupation-related morbidity patterns and their determinants among beedi workers in Murshidabad District, West Bengal. Int J Community Med Public Health. 2025 Jun 30;12(7):3202–10.
14. Varma A, Chilgod L, Bhojani U. Diverse and competing interests around tobacco: Qualitative analysis of two decades of parliamentary questions in India. BMJ Glob Health. 2021 Apr 7;6(4).
15. Glover M. Lack of Smoking Prevalence Data for Indigenous Peoples Worldwide Contributes to Epidemiological Invisibility. Data Sci J [Internet]. 2025;1. Available from: https://doi.org/10.5334/dsj-
16. Nath S, Narasimha VL, Biswas B, Kumar U, Jahnavi G, Ayub A, et al. Tobacco Use Among Tribal Communities in India: A Systematic Review and Meta-Analysis of Prevalence Studies. Cureus. 2025 Jul 14;
17. Ministry of Health & Family Welfare Government of India. [Internet]. 2024 [cited 2025 Sep 9]. Tobacco control laws and related laws in India. National Tobacco Control Program. Available from: [cited 2025 April 10]. Available from: https://ntcp.mohfw.gov.in/acts_rules_regulations
18. Ministry of Health & Family Welfare Government of India. GATS 2: Global adult tobacco survey, factsheet India 2016-17. 2017.
19. Hebbar PB, Bhojani U, Schayck O Van, Babu G, Dsouza V. Shifting the gaze on implementation : examining the association between the implementation of tobacco control laws and prevalence of tobacco using data from a nationally representative survey. BMC Public Health. 2023;1–9.

20. Institute of Public Health Bengaluru. Centre for Training, Research, and Innovation in Tribal Health (CTRITH) [Internet]. 2025. Available from: https://iphindia.org/centre-for-training-research-and-innovation-in-tribal-health-ctrith/
21. Lok Sabha (House of the People) [Internet]. 2025. General Information. Available from: https://sansad.in/ls/questions/questions
22. Rajya Sabha (Council of States) [Internet]. 2025. Introduction: Question Hour. Available from: https://sansad.in/rs/questions/general-information
23. Attride-Stirling J. Thematic networks: an analytic tool for qualitative research. Qualitative Research . 2021;1(3):385–405.
24. Dsouza R, Bhojani U. Strategic and contested use of food laws to ban smokeless tobacco products in India: a qualitative analysis of litigation. Tob Control. 2023 May 1;32(3):275–9.
25. Das SS. Traditional healing among the Tai Ahoms of Assam: changing dynamics and its interaction with the public health system. PhD Thesis. Jawaharlal Nehru University, New Delhi; 2024.
26. Witch-Hunting from a public health perspective: narratives from Assam. MPhil Thesis. Jawaharlal Nehru University, New Delhi; 2018.
27. Varma A, Chilgod L, Bhojani U. Diverse and competing interests around tobacco: Qualitative analysis of two decades of parliamentary questions in India. BMJ Glob Health. 2021;6(4):1–10.
28. Gupta PC, Asma S. Bidi smoking and public health. Ministry of Health Family Welfare. New Delhi; 2008.
29. Parliament of India. Oral answers, Question 127 [Internet]. 1987. p. 20. Available from: chrome-extension://efaidnbmnnnibpcajpcglclefindmkaj/https://eparlib.nic.in/bitstream/123456789/878354/1/08_IX_16-11-1987_p13_p15_s127.pdf
30. Government of India. Unstarred Question No: 2886 [Internet]. 2012. Available from: https://eparlib.nic.in/bitstream/123456789/616866/1/118419.pdf
31. Parliament of India (Rajya Sabha). Starred Question 11. 1993. p. 39–40.
32. Parliament of India (Rajya Sabha). Question 4940. 2000. p. 87.
33. Parliament of India (Lok Sabha). Unstarred Question 4581 [Internet]. 2016. Available from: https://eparlib.nic.in/bitstream/123456789/688010/1/40331.pdf
34. the Beedi Workers Welfare Fund Act , 1976. 1976 p. 1–7.
35. Government of India [Internet]. 1966. p. 1–15 the Beedi and Cigar Workers (Conditions of Employment) Act , 1966. Available from: https://www.indiacode.nic.in/handle/123456789/1675?sam_handle=123456789/1362
36. Parliament of India (Lok Sabha). Question 6334 [Internet]. 1995. p. 43. Available from: https://eparlib.nic.in/bitstream/123456789/2392213/1/10_XIII_19-05-1995_p26_p26_u6332.pdf
37. Parliament of India (Lok Sabha). Question 3101 [Internet]. 1995. p. 485–6. Available from: https://eparlib.nic.in/bitstream/123456789/2402879/1/10_XV_18-12-1995_p247_p247_u3101.pdf
38. Parliament of India (Lok Sabha). Question 1086 [Internet]. 1998. p. 305. Available from: https://eparlib.nic.in/bitstream/123456789/2963091/1/US_1086_02-06-1998_1.pdf

39. Parliament of India (Lok Sabha). 1995. p. 174 Question 6530. Available from: https://eparlib.nic.in/bitstream/123456789/2392411/1/10_XIII_19-05-1995_p91_p91_u6530.pdf
40. Parliament of India (Lok Sabha). Question 6332 [Internet]. 1995. p. 43. Available from: https://eparlib.nic.in/bitstream/123456789/2392213/1/10_XIII_19-05-1995_p26_p26_u6332.pdf
41. Parliament of India (Rajya Sabha). Question 572. 1990.
42. Parliament of India (Lok Sabha). Question 3321 [Internet]. 2004. Available from: https://eparlib.nic.in/bitstream/123456789/572298/1/7431.pdf
43. Parliament of India (Lok Sabha). Question 1008. 1977. p. 91.
44. Parliament of India (Lok Sabha). Question 1024 [Internet]. 1992. p. 559. Available from: https://eparlib.nic.in/bitstream/123456789/2349633/1/10_IV_14-07-1992_p287_p287_u1024.pdf
45. Parliament of India (Lok Sabha). Question 148 [Internet]. Vol. 1920. 1999. p. 34–5. Available from: https://eparlib.nic.in/bitstream/123456789/793655/1/12_IV_05031999_p21_p22_s148.pdf
46. Parliament of India (Rajya Sabha). Question 4324. 1994.
47. Parliament of India (Lok Sabha). Question 1886 [Internet]. 2002. Available from: https://eparlib.nic.in/bitstream/123456789/433857/1/48211.pdf
48. Parliament of India (Lok Sabha). Question 1886. 2002. [cited 2025 April 10] Available from: https://eparlib.nic.in/bitstream/123456789/433857/1/48211.pdf.
49. Parliament of India (Rajya Sabha). Question 572. 1990.
50. Government of India. Evaluation study on the implementation of Minimum Wages Act , 1948 in beedi making industry in Madhya Pradesh. Chandigarh; 2015.
51. Status of tendu leaf pluckers in Orissa: a study of their socio-economic conditions with special reference to children and the system of bonded labour. Delhi; 2006.
52. Rathore A, P. Ugargol A. Beedi rolling and the health hazards associated with occupational exposure: A situational review from Karnataka, India. RGUHS National Journal of Public Health. 2018;4(1).
53. Sabar B, Nayak N, Achoth L. Tendu Leaves Collection in India: Livelihoods, Rights and Challenges for Alternative to Tobacco- Evidences from Five Indian States. Journal of Governance and Public Policy. 2016;6(1):5–6.
54. Russell A, Chandra P, Robson M, Narayanan P, Joseph S, Mukherjee P, et al. Implementing FCTC Article 17 Through Participatory Research With Bidi Workers in Tamil Nadu, India. Nicotine and Tobacco Research. 2022;24(11):1714–9.
55. World Health Organization. WHO Framework Convention on Tobacco Control. Geneva; 2003.
56. Chung-Hall J, Craig L, Gravely S, Sansone N, Fong GT. Impact of the WHO FCTC over the first decade: A global evidence review prepared for the Impact Assessment Expert Group. Tob Control. 2019;28:S119–28.
57. All India Institute of Medical Sciences (Deoghar). 1st National Conference on “Tobacco Use among Tribal Communities” 20-21 July 2024. Vol. 2024. Deoghar; 2024.
58. World Health Organization. Tobacco and its environmental impact: an overview. Vol. 34. 2017.
59. Singh M, Sah P, Kumar P. Forest fires and tendu leaf collection in India: an evidence-based analysis. New Delhi; 2023.

60. Nayak NS. Tobacco Curing and Fuel Efficiency in Karnataka, India. Kathmandu; 2013.
61. SPH AIIMS Jodhpur, ICMR_NICPR, The Union. The Environmental Burden of Tobacco Products Wastes in India: tobacco use makes you ugly, your habitate uglier. 2022.
62. Ayyangar S, Jacob S. Studying the indian legislature: What does question hour reveal? Studies in Indian Politics. 2014;2(1):1–19.

(Note: the line numbers referred in this Table refers to the track-change version of the revised manuscript)

Sr. No	Comments	Authors' Responses
Editorial Comments		
1	Rationale- Kindly clarify the title - Parliamentarians' concerns about tobacco and Indigenous communities in India: a qualitative analysis of parliamentary questions (1952-2022)- is it tobacco use in indigenous communities	We would like to clarify that the paper is not focused on tobacco use among Indigenous communities. It rather aims to understand how Indian parliamentarians have framed their concerns (issues) linking tobacco and Indigenous communities in India. Interestingly, we find that the parliamentarians did not raise concerns about (high) tobacco use or its harmful health impacts among Indigenous communities in general. Instead, major concerns they raised were related to Indigenous community members working in tobacco (esp. Bidi) supply chains highlighting, occupational hazards, exploitative work conditions, poor access to healthcare services and negative impact of trade/investment policies on tobacco-dependent livelihoods among Indigenous communities. To bring more clarity, we have made a minor edit in the Title:<br><br>"Policymakers' concerns linking tobacco and Indigenous communities in India: a qualitative analysis of parliamentary questions (1952-2022)"
2	Please consider improving your rationale. do you think parliament would see indigenous communities different from rest of the country while discussing tobacco. if yes, this can be added in the text. and also, why this segregated review particularly for indigenous communities would be useful and how overall results may vary and	Thank you! In past, we have analyzed Indian parliamentarians' concerns related to tobacco in general (not specific to Indigenous communities) and we did notice that concerns about tobacco-related health harms and the need for tobacco control policies were becoming dominant in the last couple of decades. However, we felt that exploring parliamentarians' concerns linking tobacco and Indigenous communities would be crucial first step to understand why there is no specific policy/program focused on addressing high tobacco use among Indigenous communities and the growing social disparity in tobacco use. We presumed that parliamentarians would be concerned about Indigenous community because the Indian constitution recognizes them as specifically vulnerable groups that have suffered social injustice and provides for constitutional safeguards for their development. In fact, the Government of India has a specific Ministry of Tribal Affairs. We have now revised the text under Introduction section incorporating these points:

	so our interpretation if whole population is considered.	See lines: 97-117:<br><br>"There have been several policy initiatives by the Government of India for reducing tobacco use(11) and these initiatives seem to be making some positive impact.(12, 13). However, there is no specific focus or considerations for Indigenous communities within these initiatives."… "As parliamentarians play a crucial role in shaping policy discourse and policymaking, including for tobacco control, we aimed to study how parliamentarians in India framed their concerns related to tobacco and indigenous communities in India over time. Our earlier analysis of parliamentarians' concerns related to tobacco in general - not specific to Indigenous communities - indicated that parliamentarians were increasingly concerned about health harms of tobacco and the need for tobacco control measures amidst continuing concerns about tobacco-linked revenues and livelihoods.(15) We presumed that parliamentarians will be interested in issues affecting Indigenous communities given they are considered special groups who suffered historical injustice and who have constitutional safeguards.(10) In fact, there is a separate Ministry of Tribal Affairs within the Government of India. Hence, we aimed to better understand how parliamentarians framed their concerns linking tobacco and Indigenous communities over time."
3	line 99- please consider sharing a direct link to the data depository or cite it for ease of other readers.	Thank you! We have now provided citations leading to the digital, searchable data depositories (question and answer portals of both houses of Indian parliament).<br><br>See lines: 190-193:<br>"We searched the publicly available digital catalogues of parliamentary questions from both houses; Lok Sabha (Lower House)(19) and Rajya Sabha (Upper House)(20) separately using a combination of search terms 'tobacco' and 'tribal' within the full text of the transcripts without any search filters."<br>19. Loksabha. Questions and Answers. Available from: https://sansad.in/ls/questions/questions-and-answers<br>20. Rajyasabha. Questions and Answers. Available from: https://sansad.in/rs/questions/questions-and-answers
4	table1: Please consider making table less complicated. I don't	Thank you! We have improved the readability of the table by putting in percentages (apart from the absolute number). We believe that this table helps readers with a few important attributes of the questions

	know how international audience would benefit from such table and data. maybe u can consider in power and in opposition or something like that. is this table also pointing towards TII. Less questions means more vested interest. - this is my view of the things- different people may think differently and paper then conveys a different meaning.	(primary data source for this paper) including the proportion of MPs coming from Indigenous communities asking these questions, giving readers a sense of (very limited) direct representation of Indigenous voices in these questions. It is difficult to indicate weather MPs asking these questions belonged to the ruling or opposition parties, as they would have possibly belonged to both these categories given the questions cover a long period of time (1952-2022). Typically, most questions at any given point in time are asked by the MPs from opposition parties to the ruling governments. We see the logic of you considering the number of questions asked as a possible indicator of tobacco industry interference. However, it is difficult to interpret it this way because many factors are likely to shape an MP's decision to ask a question, including his/her social identity (Indigenous status), his/her awareness/interest in tobacco/Indigenous community issues among others. Hence, we have refrained from making such a connection. I hope that is acceptable to you.<br><br>See the revised Table 1 (line 296).
5	Discussion- Consider adding a para on the direct and indirect policy implications of the study. and also on emerging recommendations from the study.	Thanks for this suggestion. We have now added a couple of paragraphs in the Discussion section highlighting specific policy/program implications including a line on the future relevant research.<br><br>See lines: 746-773:<br>"Our findings and their discussion above have some implications for actions and further research related to tobacco control among Indigenous communities in India. Overall, it highlights the limited but diverse political concerns linking tobacco and Indigenous communities in India. We find that among the Indian Parliamentarians, but also in tobacco control discourse in general, there is very limited recognition of health consequences from the high overall tobacco use among many Indigenous communities, and the growing social disparity in tobacco use. It is desirable to develop a special focus on indigenous communities within tobacco control efforts, making such efforts relevant, acceptable, and informed by voices from indigenous communities. In this respect, we have seen a few examples in recent past including a webinar series and the first-of-its-kind national conference on tobacco use among tribal communities by the All India Institute of Medical Sciences (Deoghar)(56) as well as the efforts at understanding historical and socio-cultural pathways of substance use and creating context-specific tobacco-cessation support among indigenous communities through the Centre for Training, Research and Innovations in Tribal Health(14). However, we

		need to do more including enhanced implementation, using context-specific strategies, of the national tobacco control program in districts with Indigenous communities.<br><br>The other important implication is to check the prevailing labour exploitation of Indigenous community workers, especially in bidi supply chains, while strengthening efforts in providing alternative (non-tobacco) livelihoods options that are safer and viable. While there have been efforts to promote alternative crops in place of tobacco, such efforts have specifically targeted growers of cigarette tobacco.(57) There do not seem to be major efforts from governments to promote alternatives for marginal workers in bidi industry. Prioritising them under the prevailing wage employment promotion initiatives like the Mahatma Gandhi Rural Employment Guarantee Scheme(58) and the Pradhan Mantri Kaushal Vikas Yojana (Skill India Mission)(59) can help in this direction. While our study was limited to parliamentary concerns, the future studies could look at other sources (such as news media, social movements, civil society voices) to map the broader public/policy discourse linking tobacco and Indigenous communities in India. It is time that the public health research and advocacy community expand their remit to engage with complex and multiple ways in which tobacco use, and the tobacco industry are impacting indigenous communities to make any positive difference."
First Reviewer's Comments		
6	My overarching suggestion is that the paper should adopt a more outward-facing perspective. Specifically, it would benefit from linking its findings more clearly to the broader literature, both within India and internationally. While the study adds new knowledge, it does not yet fully situate its findings within existing gaps in the literature for a global audience.<br>In the introduction, it would be helpful for the authors to provide	Thank for this suggestion! We have now added more contextual information in the Introduction section and, in general, ensured linking our work to the broader literature. Please see the revisions made to the Introduction section – lines: 69-96:<br><br>"This concern about high level of tobacco use among indigenous communities is not limited to India. Studies have found that in several countries, especially in Australia, New Zealand, United States of America and Canada, the prevalence of commercial tobacco use among Indigenous communities has been much higher compared to non-Indigenous communities.(4) Tobacco industry has been recognised as one of the important commercial determinants affecting health and wellbeing of Indigenous communities.(5) Many countries have recognised this and have developed specific strategies to reduce such disparities.(6) For example, Australia, in its National Drug Strategy (2017-2026) identifies Aboriginal and Torres Strait Islanders people as priority groups with a specific sub-strategy for these communities indicating need for careful, contextual and participative interventions.(7) Canada's Tobacco

	international readers with more context about indigenous communities in India. A general overview would support a better understanding of the scale and nature of the issues discussed, and allow for comparisons with indigenous populations in other parts of the world who often face similar challenges.	Strategy prioritises Indigenous communities ensuring specific funding to First Nations, Inuits, and Metis Nation people enabling development of their own plans to address commercial tobacco use.(8)<br><br>India is home to 7305 ethnic groups that are officially notified as ‘Scheduled Tribes’. According to the Census of India (2011), 104.5 million people belonged to the ‘Scheduled Tribe’ category constituting 8.6 percent of the total population in India.(9) Indigenous communities in India are highly diverse in terms of culture, language, social organization, and their habitat. In general, Indigenous communities have low rates of literacy, access to health infrastructure and poor nutrition and health indicators.(9) There are constitutional safeguards to protect and promote rights of Scheduled Tribes including reservations in higher education and election seats.(10)”
7	The background section could be strengthened by including information about any tobacco control laws or policies that specifically address indigenous populations. For example, are there any tobacco control campaigns or cessation services that target these communities? Even noting the absence of such initiatives would help to contextualise the study's findings.	Thanks! Unfortunately, there are no tobacco control programs/policies (any major documented ones) that are primarily meant for or prioritizes Indigenous communities. We have now mentioned the lack of such specific initiatives in the Introduction section.<br><br>See lines: 97-100:<br>“There have been several policy initiatives by the Government of India for reducing tobacco use(11) and these initiatives seem to be making some positive impact.(12, 13). However, there is no specific focus or considerations for Indigenous communities within these initiatives.”
8	The paper should also consider the issue of tobacco surveillance. Are indigenous populations included in surveys like GATS or GYTS? Are there any national surveys beyond	Thanks! GATS does mention about the Scheduled Tribe category in its sampling consideration and has one question related to this in the main questionnaire. However, GATS factsheet and reports do not separately provide data about tobacco use among Indigenous communities. GATS does not have Scheduled Tribe as a category. We have referenced the data from other nationally representative surveys that could be used to surveil tobacco use among Indigenous communities (i.e., National Family Health Surveys; National Sample Surveys).

	the report already referenced that include relevant data?	
9	Additionally, if available, information about tobacco industry marketing strategies targeting indigenous populations would be valuable in the background section.	Thanks! To our knowledge, no such specific information available in scientific or gray literature about tobacco industry marketing strategies targeting Indigenous communities in India. We have, in past, researched tobacco industry strategies in India and have not come across such information.
10	Regarding the methods, it would be useful to clarify whether the transcripts analysed included parliamentary debates on specific tobacco control bills or related legislation. If such transcripts were omitted or unavailable, what impact does this have on the study? This could be addressed as a potential limitation	Thanks! While we included transcripts of all the question types related to tobacco and indigenous communities, we have not analysed 'debates', typically long-form transactions that happen around major legislative developments. Though it is less likely that debates would have offered additional data given the tobacco-related legislations/programs do not have specific focus on Indigenous communities, we have now added this to the limitation within the Discussion section.<br><br>See lines: 798-802:<br><br>"While we included all the question types, we did not include the debates – a longform transaction specifically around legislative developments. While it is a limitation, it is also less likely that it would have added any substantive data given that tobacco-related legislations do not have specific emphasis on Indigenous communities."
11	It is also unclear how the inclusion or exclusion of transcripts was handled. Did two authors independently screen and select the transcripts, or did one author do so with subsequent review by the second? How were disagreements resolved? Depending on the process, this	Thanks! We now clarified this process under the Methods section.<br><br>See lines: 200-208:<br>"The first author (SSD) read all the transcripts in full and further excluded those that did not contain at least one of the two categories of search terms (tobacco and tribal) or had these terms occurring incidentally (often in answers) while the transcript had no substantive relevance to the subject of our study. Fig 1 provides details about the sourcing and screening outcomes. The first author then discussed the shortlisted questions as well as potential exclusion that lacked clarity with the second author (UB) and

	could be discussed as either a limitation or a strength.	made a decision about inclusion/exclusion based on consensus. In case of disagreement, the matter was referred to the third author (PNS), who discussed with the first two authors and made a final decision."
12	As someone who is not a qualitative researcher, I found the description of the analytical approach unclear. Why did the authors not consider using framework analysis, which is known for its flexibility? While the research may be novel, parliamentary records have previously been analysed in the context of tobacco control. It is not evident whether the authors consulted prior studies of this kind. Providing more detail on the analytic method and referencing specific qualitative approaches would help clarify this.	Thanks! While we have cited a method paper for the thematic content analysis, we have not used (and referenced) any specific framework because such framework (to analyze parliamentary questions OR to link tobacco and Indigenous communities) does not exist. The only earlier work analyzing parliamentary questions in context of tobacco by the same authors was not aimed at creating a framework. Given the lack of useful framework/model and prior studies linking tobacco and Indigenous communities (beyond epidemiological inquiries), we preferred to keep the inquiry exploratory using inductive and open coding guided by the study objective.
13	The dataset spans a lengthy period of 70 years. However, the paper does not currently contextualise the data in relation to the timeline of tobacco control laws and policies in India. Temporal context matters: without knowing what else was happening when these issues were raised, the findings	Thanks for this useful suggestion! We have now added a schematic (Figure 3) that provides an overview of relation between major tobacco control legislation (or programs) and tobacco-related parliamentary questions. We added a few lines interpreting this relationship in the Results section.<br><br>See lines: 284-294:<br>"Nearly three fourth (73.8%) of the total questions (analysed in this study) were asked in post 1990 period that typically saw frequent tobacco control reforms. In general, questions spiked preceding major tobacco-related reforms (see Fig 3)."

<table>
<tr><td></td><td>risk being presented in isolation. A timeline covering 1952–2022, showing when the analysed transcripts were produced and when key tobacco control laws were introduced, would be very helpful.</td><td>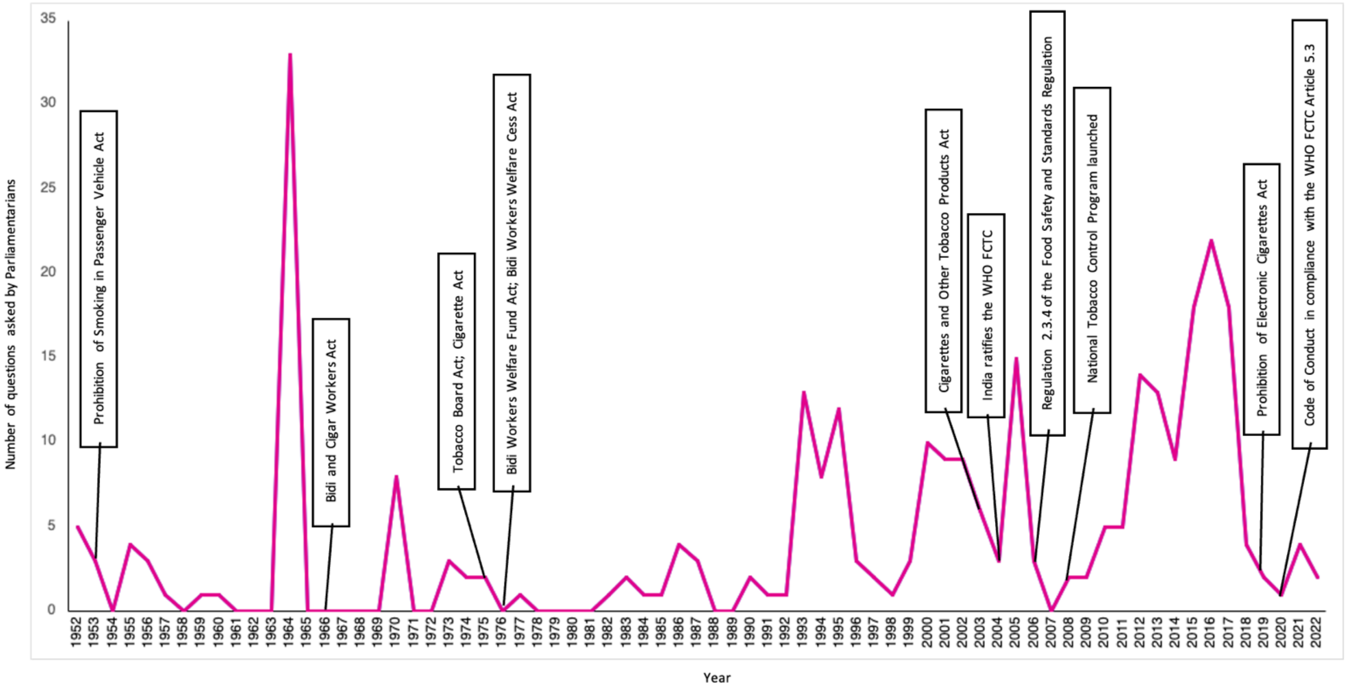
</td></tr>
<tr><td>14</td><td>The discussion section does not adequately link the findings to existing literature, either from India or internationally. Indigenous communities around the world often experience high levels of tobacco use and related harms. It would be useful to know whether the findings of this study</td><td>Thank you for this comment. While we have now added a paragraph relating our study to other international contexts (Indigenous communities in Australia, Canada, Unites States of America) in the Introduction section (see the earlier Sr No 6 in this tables). In the results section, we have cited other studies corroborating the parliamentarians' concerns (especially around exploitation of bidi and tendu workers; and environmental impact of tobacco) or contrasting (with earlier work on tobacco related parliamentarians' concerns in general).</td></tr>
</table>

	corroborate, expand upon, or contradict existing research.	
15	The limitations section should focus on the limitations of the study design or methodology rather than the data itself. For instance, if the transcripts contain few voices of women or indigenous parliamentarians, that is an important finding in its own right—not a limitation.	Thanks! We have now added limitations specific to the design/methods used.<br><br>See lines: 798-808:<br>"While we included all the question types, we did not include the debates – a longform transaction specifically around legislative developments. While it is a limitation, it is also less likely that it would have added any substantive data given that tobacco-related legislations do not have specific emphasis on Indigenous communities.<br><br>The parliamentary questions are a limited component of the broader policy discourse. Furthermore. parliamentary questions are typically factual and concise devoid of candid details. These questions are often framed with many considerations (like party agenda, political signalling, personal priority, individual interests) and do not always reflect genuine concerns of constituents."
16	Finally, the authors could strengthen the paper by proposing specific actions that should follow from their findings. What are the implications for policy? How should tobacco control laws or programmes be adapted in light of these insights? What recommendations can be made to parliamentarians or other stakeholders? The authors have identified important gaps—such as the neglect of indigenous health and environmental concerns—but	Thanks! We have now revised the Discussion section adding policy/programmatic implications as well as need for the future research. (See the earlier Sr No 5 in this table)

	they stop short of offering concrete suggestions for change.	
Second Reviewer's Comments		
17	Was alternate livelihood used as a key word?	No, terms related to alternative livelihoods were not used in search strategy. However, our search strategy basically used combination of various terms used to refer to tobacco and Indigenous communities – and hence, captured Parliamentarians' questions related to livelihoods.
18	In the discussion, it is mentioned that health concerns were not raised. But in results section there is discussion about tuberculosis. Please clarify. The sentence can be modified accordingly.	Thanks for pointing this! What we meant in the discussion is that whenever parliamentarians expressed health concerns (including Tuberculosis, hunger etc.), they typically did this in context of Indigenous community members as workers in Bidi (tobacco) sector. We have edited one of the sentences to avoid confusion.<br><br>See lines: 700-701:<br>"We did not come across any concerns about tobacco use and its health harms among indigenous communities in general, beyond as workers in tobacco sector."
