# Supplementary material for: Policymakers’ concerns linking tobacco and Indigenous communities in India: A qualitative analysis of parliamentary questions (1952–2022)
Source: PLOS Glob Public Health. 2026 Feb 24;6(2):e0004601. doi: 10.1371/journal.pgph.0004601 (PMC12931741; doi:10.1371/journal.pgph.0004601)
Supplement: Attachment — Submitted filename: peer review_responses.docx [file pgph.0004601.s003.docx]

(Note: the line numbers referred in this Table refers to the track-change version of the revised manuscript)

| Sr.  No | Comments | Authors’ Responses |
| --- | --- | --- |
| Editorial Comments | | |
| 1 | Rationale- Kindly clarify the title - Parliamentarians’ concerns about tobacco and Indigenous communities in India: a qualitative analysis of parliamentary questions (1952-2022)- is it tobacco use in indigenous communities | We would like to clarify that the paper is not focused on tobacco use among Indigenous communities. It rather aims to understand how Indian parliamentarians have framed their concerns (issues) linking tobacco and Indigenous communities in India. Interestingly, we find that the parliamentarians did not raise concerns about (high) tobacco use or its harmful health impacts among Indigenous communities in general. Instead, major concerns they raised were related to Indigenous community members working in tobacco (esp. Bidi) supply chains highlighting, occupational hazards, exploitative work conditions, poor access to healthcare services and negative impact of trade/investment policies on tobacco-dependent livelihoods among Indigenous communities. To bring more clarity, we have made a minor edit in the Title:  “Policymakers’ concerns linking tobacco and Indigenous communities in India: a qualitative analysis of parliamentary questions (1952-2022)” |
| 2 | Please consider improving your rationale. do you think parliament would see indigenous communities different from rest of the country while discussing tobacco. if yes, this can be added in the text. and also, why this segregated review particularly for indigenous communities would be useful and how overall results may vary and so our interpretation if whole population is considered. | Thank you! In past, we have analyzed Indian parliamentarians’ concerns related to tobacco in general (not specific to Indigenous communities) and we did notice that concerns about tobacco-related health harms and the need for tobacco control policies were becoming dominant in the last couple of decades. However, we felt that exploring parliamentarians’ concerns linking tobacco and Indigenous communities would be crucial first step to understand why there is no specific policy/program focused on addressing high tobacco use among Indigenous communities and the growing social disparity in tobacco use. We presumed that parliamentarians would be concerned about Indigenous community because the Indian constitution recognizes them as specifically vulnerable groups that have suffered social injustice and provides for constitutional safeguards for their development. In fact, the Government of India has a specific Ministry of Tribal Affairs. We have now revised the text under Introduction section incorporating these points:  See lines: 97-117:  “There have been several policy initiatives by the Government of India for reducing tobacco use(11) and these initiatives seem to be making some positive impact.(12, 13). However, there is no specific focus or considerations for Indigenous communities within these initiatives.”… “As parliamentarians play a crucial role in shaping policy discourse and policymaking, including for tobacco control, we aimed to study how parliamentarians in India framed their concerns related to tobacco and indigenous communities in India over time. Our earlier analysis of parliamentarians’ concerns related to tobacco in general - not specific to Indigenous communities - indicated that parliamentarians were increasingly concerned about health harms of tobacco and the need for tobacco control measures amidst continuing concerns about tobacco-linked revenues and livelihoods.(15) We presumed that parliamentarians will be interested in issues affecting Indigenous communities given they are considered special groups who suffered historical injustice and who have constitutional safeguards.(10) In fact, there is a separate Ministry of Tribal Affairs within the Government of India. Hence, we aimed to better understand how parliamentarians framed their concerns linking tobacco and Indigenous communities over time.” |
| 3 | line 99- please consider sharing a direct link to the data depository or cite it for ease of other readers. | Thank you! We have now provided citations leading to the digital, searchable data depositories (question and answer portals of both houses of Indian parliament).  See lines: 190-193:  “We searched the publicly available digital catalogues of parliamentary questions from both houses; Lok Sabha (Lower House)(19) and Rajya Sabha (Upper House)(20) separately using a combination of search terms ‘tobacco’ and ‘tribal’ within the full text of the transcripts without any search filters.”  19. Loksabha. Questions and Answers. Available from: <https://sansad.in/ls/questions/questions-and-answers>  20. Rajyasabha. Questions and Answers. Available from: <https://sansad.in/rs/questions/questions-and-answers> |
| 4 | table1: Please consider making table less complicated. I don’t know how international audience would benefit from such table and data. maybe u can consider in power and in opposition or something like that. is this table also pointing towards TII. Less questions means more vested interest. - this is my view of the things- different people may think differently and paper then conveys a different meaning. | Thank you! We have improved the readability of the table by putting in percentages (apart from the absolute number). We believe that this table helps readers with a few important attributes of the questions (primary data source for this paper) including the proportion of MPs coming from Indigenous communities asking these questions, giving readers a sense of (very limited) direct representation of Indigenous voices in these questions. It is difficult to indicate weather MPs asking these questions belonged to the ruling or opposition parties, as they would have possibly belonged to both these categories given the questions cover a long period of time (1952-2022). Typically, most questions at any given point in time are asked by the MPs from opposition parties to the ruling governments. We see the logic of you considering the number of questions asked as a possible indicator of tobacco industry interference. However, it is difficult to interpret it this way because many factors are likely to shape an MP’s decision to ask a question, including his/her social identity (Indigenous status), his/her awareness/interest in tobacco/Indigenous community issues among others. Hence, we have refrained from making such a connection. I hope that is acceptable to you.  See the revised Table 1 (line 296). |
| 5 | Discussion- Consider adding a para on the direct and indirect policy implications of the study. and also on emerging recommendations from the study. | Thanks for this suggestion. We have now added a couple of paragraphs in the Discussion section highlighting specific policy/program implications including a line on the future relevant research.  See lines: 746-773:  “Our findings and their discussion above have some implications for actions and further research related to tobacco control among Indigenous communities in India. Overall, it highlights the limited but diverse political concerns linking tobacco and Indigenous communities in India. We find that among the Indian Parliamentarians, but also in tobacco control discourse in general, there is very limited recognition of health consequences from the high overall tobacco use among many Indigenous communities, and the growing social disparity in tobacco use. It is desirable to develop a special focus on indigenous communities within tobacco control efforts, making such efforts relevant, acceptable, and informed by voices from indigenous communities. In this respect, we have seen a few examples in recent past including a webinar series and the first-of-its-kind national conference on tobacco use among tribal communities by the All India Institute of Medical Sciences (Deoghar)(56) as well as the efforts at understanding historical and socio-cultural pathways of substance use and creating context-specific tobacco-cessation support among indigenous communities through the Centre for Training, Research and Innovations in Tribal Health(14). However, we need to do more including enhanced implementation, using context-specific strategies, of the national tobacco control program in districts with Indigenous communities.  The other important implication is to check the prevailing labour exploitation of Indigenous community workers, especially in bidi supply chains, while strengthening efforts in providing alternative (non-tobacco) livelihoods options that are safer and viable. While there have been efforts to promote alternative crops in place of tobacco, such efforts have specifically targeted growers of cigarette tobacco.(57) There do not seem to be major efforts from governments to promote alternatives for marginal workers in bidi industry. Prioritising them under the prevailing wage employment promotion initiatives like the Mahatma Gandhi Rural Employment Guarantee Scheme(58) and the Pradhan Mantri Kaushal Vikas Yojana (Skill India Mission)(59) can help in this direction. While our study was limited to parliamentary concerns, the future studies could look at other sources (such as news media, social movements, civil society voices) to map the broader public/policy discourse linking tobacco and Indigenous communities in India. It is time that the public health research and advocacy community expand their remit to engage with complex and multiple ways in which tobacco use, and the tobacco industry are impacting indigenous communities to make any positive difference.” |
| First Reviewer’s Comments | | |
| 6 | My overarching suggestion is that the paper should adopt a more outward-facing perspective. Specifically, it would benefit from linking its findings more clearly to the broader literature, both within India and internationally. While the study adds new knowledge, it does not yet fully situate its findings within existing gaps in the literature for a global audience. In the introduction, it would be helpful for the authors to provide international readers with more context about indigenous communities in India. A general overview would support a better understanding of the scale and nature of the issues discussed, and allow for comparisons with indigenous populations in other parts of the world who often face similar challenges. | Thank for this suggestion! We have now added more contextual information in the Introduction section and, in general, ensured linking our work to the broader literature. Please see the revisions made to the Introduction section – lines: 69-96:  “This concern about high level of tobacco use among indigenous communities is not limited to India. Studies have found that in several countries, especially in Australia, New Zealand, United States of America and Canada, the prevalence of commercial tobacco use among Indigenous communities has been much higher compared to non-Indigenous communities.(4) Tobacco industry has been recognised as one of the important commercial determinants affecting health and wellbeing of Indigenous communities.(5) Many countries have recognised this and have developed specific strategies to reduce such disparities.(6) For example, Australia, in its National Drug Strategy (2017-2026) identifies Aboriginal and Torres Strait Islanders people as priority groups with a specific sub-strategy for these communities indicating need for careful, contextual and participative interventions.(7) Canada’s Tobacco Strategy prioritises Indigenous communities ensuring specific funding to First Nations, Inuits, and Metis Nation people enabling development of their own plans to address commercial tobacco use.(8)  India is home to 7305 ethnic groups that are officially notified as ‘Scheduled Tribes’. According to the Census of India (2011), 104.5 million people belonged to the ‘Scheduled Tribe’ category constituting 8.6 percent of the total population in India.(9) Indigenous communities in India are highly diverse in terms of culture, language, social organization, and their habitat. In general, Indigenous communities have low rates of literacy, access to health infrastructure and poor nutrition and health indicators.(9) There are constitutional safeguards to protect and promote rights of Scheduled Tribes including reservations in higher education and election seats.(10)” |
| 7 | The background section could be strengthened by including information about any tobacco control laws or policies that specifically address indigenous populations. For example, are there any tobacco control campaigns or cessation services that target these communities? Even noting the absence of such initiatives would help to contextualise the study's findings. | Thanks! Unfortunately, there are no tobacco control programs/policies (any major documented ones) that are primarily meant for or prioritizes Indigenous communities. We have now mentioned the lack of such specific initiatives in the Introduction section.  See lines: 97-100:  “There have been several policy initiatives by the Government of India for reducing tobacco use(11) and these initiatives seem to be making some positive impact.(12, 13). However, there is no specific focus or considerations for Indigenous communities within these initiatives.” |
| 8 | The paper should also consider the issue of tobacco surveillance. Are indigenous populations included in surveys like GATS or GYTS? Are there any national surveys beyond the report already referenced that include relevant data? | Thanks! GATS does mention about the Scheduled Tribe category in its sampling consideration and has one question related to this in the main questionnaire. However, GATS factsheet and reports do not separately provide data about tobacco use among Indigenous communities. GATS does not have Scheduled Tribe as a category. We have referenced the data from other nationally representative surveys that could be used to surveil tobacco use among Indigenous communities (i.e., National Family Health Surveys; National Sample Surveys). |
| 9 | Additionally, if available, information about tobacco industry marketing strategies targeting indigenous populations would be valuable in the background section. | Thanks! To our knowledge, no such specific information available in scientific or gray literature about tobacco industry marketing strategies targeting Indigenous communities in India. We have, in past, researched tobacco industry strategies in India and have not come across such information. |
| 10 | Regarding the methods, it would be useful to clarify whether the transcripts analysed included parliamentary debates on specific tobacco control bills or related legislation. If such transcripts were omitted or unavailable, what impact does this have on the study? This could be addressed as a potential limitation | Thanks! While we included transcripts of all the question types related to tobacco and indigenous communities, we have not analysed ‘debates’, typically long-form transactions that happen around major legislative developments. Though it is less likely that debates would have offered additional data given the tobacco-related legislations/programs do not have specific focus on Indigenous communities, we have now added this to the limitation within the Discussion section.  See lines: 798-802:  “While we included all the question types, we did not include the debates – a longform transaction specifically around legislative developments. While it is a limitation, it is also less likely that it would have added any substantive data given that tobacco-related legislations do not have specific emphasis on Indigenous communities.” |
| 11 | It is also unclear how the inclusion or exclusion of transcripts was handled. Did two authors independently screen and select the transcripts, or did one author do so with subsequent review by the second? How were disagreements resolved? Depending on the process, this could be discussed as either a limitation or a strength. | Thanks! We now clarified this process under the Methods section.  See lines: 200-208: “The first author (SSD) read all the transcripts in full and further excluded those that did not contain at least one of the two categories of search terms (tobacco and tribal) or had these terms occurring incidentally (often in answers) while the transcript had no substantive relevance to the subject of our study. Fig 1 provides details about the sourcing and screening outcomes. The first author then discussed the shortlisted questions as well as potential exclusion that lacked clarity with the second author (UB) and made a decision about inclusion/exclusion based on consensus. In case of disagreement, the matter was referred to the third author (PNS), who discussed with the first two authors and made a final decision.” |
| 12 | As someone who is not a qualitative researcher, I found the description of the analytical approach unclear. Why did the authors not consider using framework analysis, which is known for its flexibility? While the research may be novel, parliamentary records have previously been analysed in the context of tobacco control. It is not evident whether the authors consulted prior studies of this kind. Providing more detail on the analytic method and referencing specific qualitative approaches would help clarify this. | Thanks! While we have cited a method paper for the thematic content analysis, we have not used (and referenced) any specific framework because such framework (to analyze parliamentary questions OR to link tobacco and Indigenous communities) does not exist. The only earlier work analyzing parliamentary questions in context of tobacco by the same authors was not aimed at creating a framework. Given the lack of useful framework/model and prior studies linking tobacco and Indigenous communities (beyond epidemiological inquiries), we preferred to keep the inquiry exploratory using inductive and open coding guided by the study objective. |
| 13 | The dataset spans a lengthy period of 70 years. However, the paper does not currently contextualise the data in relation to the timeline of tobacco control laws and policies in India. Temporal context matters: without knowing what else was happening when these issues were raised, the findings risk being presented in isolation. A timeline covering 1952–2022, showing when the analysed transcripts were produced and when key tobacco control laws were introduced, would be very helpful. | Thanks for this useful suggestion! We have now added a schematic (Figure 3) that provides an overview of relation between major tobacco control legislation (or programs) and tobacco-related parliamentary questions. We added a few lines interpreting this relationship in the Results section.  See lines: 284-294:  “Nearly three fourth (73.8%) of the total questions (analysed in this study) were asked in post 1990 period that typically saw frequent tobacco control reforms. In general, questions spiked preceding major tobacco-related reforms (see Fig 3).”   |
| 14 | The discussion section does not adequately link the findings to existing literature, either from India or internationally. Indigenous communities around the world often experience high levels of tobacco use and related harms. It would be useful to know whether the findings of this study corroborate, expand upon, or contradict existing research. | Thank you for this comment. While we have now added a paragraph relating our study to other international contexts (Indigenous communities in Australia, Canada, Unites States of America) in the Introduction section (see the earlier Sr No 6 in this tables). In the results section, we have cited other studies corroborating the parliamentarians’ concerns (especially around exploitation of bidi and tendu workers; and environmental impact of tobacco) or contrasting (with earlier work on tobacco related parliamentarians’ concerns in general). |
| 15 | The limitations section should focus on the limitations of the study design or methodology rather than the data itself. For instance, if the transcripts contain few voices of women or indigenous parliamentarians, that is an important finding in its own right—not a limitation. | Thanks! We have now added limitations specific to the design/methods used.  See lines: 798-808:  “While we included all the question types, we did not include the debates – a longform transaction specifically around legislative developments. While it is a limitation, it is also less likely that it would have added any substantive data given that tobacco-related legislations do not have specific emphasis on Indigenous communities.  The parliamentary questions are a limited component of the broader policy discourse. Furthermore. parliamentary questions are typically factual and concise devoid of candid details. These questions are often framed with many considerations (like party agenda, political signalling, personal priority, individual interests) and do not always reflect genuine concerns of constituents.” |
| 16 | Finally, the authors could strengthen the paper by proposing specific actions that should follow from their findings. What are the implications for policy? How should tobacco control laws or programmes be adapted in light of these insights? What recommendations can be made to parliamentarians or other stakeholders? The authors have identified important gaps—such as the neglect of indigenous health and environmental concerns—but they stop short of offering concrete suggestions for change. | Thanks! We have now revised the Discussion section adding policy/programmatic implications as well as need for the future research. (See the earlier Sr No 5 in this table) |
| Second Reviewer’s Comments | | |
| 17 | Was alternate livelihood used as a key word? | No, terms related to alternative livelihoods were not used in search strategy. However, our search strategy basically used combination of various terms used to refer to tobacco and Indigenous communities – and hence, captured Parliamentarians’ questions related to livelihoods. |
| 18 | In the discussion, it is mentioned that health concerns were not raised. But in results section there is discussion about tuberculosis. Please clarify. The sentence can be modified accordingly. | Thanks for pointing this! What we meant in the discussion is that whenever parliamentarians expressed health concerns (including Tuberculosis, hunger etc.), they typically did this in context of Indigenous community members as workers in Bidi (tobacco) sector. We have edited one of the sentences to avoid confusion.  See lines: 700-701:  “We did not come across any concerns about tobacco use and its health harms among indigenous communities in general, beyond as workers in tobacco sector.” |
