# Supplementary material for: Policymakers’ concerns linking tobacco and Indigenous communities in India: A qualitative analysis of parliamentary questions (1952–2022)
Source: PLOS Glob Public Health. 2026 Feb 24;6(2):e0004601. doi: 10.1371/journal.pgph.0004601 (PMC12931741; doi:10.1371/journal.pgph.0004601)
Supplement: Attachment — Submitted filename: Response to Reviewers.docx [file pgph.0004601.s004.docx]

Dear Editor,

PLoS Global Public Health

Many thanks for getting our manuscript further reviewed and providing us with the reviewer’s comments. We have now revised our manuscript. In the table below, we provide a point-by-point response to the reviewer’s comments. We have also copy-edited our manuscript for spelling/grammar corrections. We hope that the revised manuscript meets your expectations for publication in the PLoS Global Public Health.

Warm regards,

Dr Upendra Bhojani

| Sr. no. | Reviewer’s comments | Authors’ response |
| --- | --- | --- |
| 1 | Nath S, Narasimha VL, Biswas B, Kumar U, Jahnavi G, Ayub A, Alam B, Jha N, Venugopal V, Bhattacharya S, Singh RJ, Varshney S. Tobacco Use Among Tribal Communities in India: A Systematic Review and Meta-Analysis of Prevalence Studies. Cureus. 2025 Jul 14;17(7):e87874. doi: 10.7759/cureus.87874. PMID: 40809626; PMCID: PMC12344541.Authors can quote the latest pooled prevalence data of tobacco use in tribal population from the SRMA mentioned above. | Thank you for this suggestion. We have now added these latest findings in the Introduction section, adding the reference.  “Another recent effort at meta-analysis of 39 studies involving 56,883 individuals from Indigenous communities across India estimated a pooled prevalence of tobacco use at 60% (66% among men, and 42% among women).(4)” |
| 2 | Lines 169-174: “Both these authors had formal training in qualitative research. The first author has worked with Indigenous communities for her doctoral and post-doctoral work.(22,23) The last author has been researching tobacco control for about two decades including published work using parliamentary questions.(15) He has been collaborating with scholars working with Indigenous communities including some of the members from the Indigenous community”  This entire section can be removed, and can be included in the author contribution section, wherein a statement of the authors' expertise in this domain can be mentioned. | While we agree that this paragraph could also fit in the ‘authors’ contribution’ section, we prefer to keep it here as the intention is to inform researchers’ positionality (hence, the narrative about their overall expertise and engagement with Indigenous communities beyond just the contribution to this particular study). We hope that is acceptable. |
